# ScanFold: an approach for genome-wide discovery of local RNA structural elements—applications to Zika virus and HIV

**DOI:** 10.7717/peerj.6136

**Published:** 2018-12-18

**Authors:** Ryan J. Andrews, Julien Roche, Walter N. Moss

**Affiliations:** Roy J. Carver Department of Biophysics, Biochemistry and Molecular Biology, Iowa State University, Ames, IA, USA

**Keywords:** RNA, RNA structure, Zika virus, Motif discovery, Bioinformatics, Sequence analysis, ncRNA, HIV

## Abstract

In addition to encoding RNA primary structures, genomes also encode RNA secondary and tertiary structures that play roles in gene regulation and, in the case of RNA viruses, genome replication. Methods for the identification of functional RNA structures in genomes typically rely on scanning analysis windows, where multiple partially-overlapping windows are used to predict RNA structures and folding metrics to deduce regions likely to form functional structure. Separate structural models are produced for each window, where the step size can greatly affect the returned model. This makes deducing unique local structures challenging, as the same nucleotides in each window can be alternatively base paired. We are presenting here a new approach where all base pairs from analysis windows are considered and weighted by favorable folding. This results in unique base pairing throughout the genome and the generation of local regions/structures that can be ranked by their propensity to form unusually thermodynamically stable folds. We applied this approach to the Zika virus (ZIKV) and HIV-1 genomes. ZIKV is linked to a variety of neurological ailments including microcephaly and Guillain–Barré syndrome and its (+)-sense RNA genome encodes two, previously described, functionally essential structured RNA regions. HIV, the cause of AIDS, contains multiple functional RNA motifs in its genome, which have been extensively studied. Our approach is able to successfully identify and model the structures of known functional motifs in both viruses, while also finding additional regions likely to form functional structures. All data have been archived at the RNAStructuromeDB (www.structurome.bb.iastate.edu), a repository of RNA folding data for humans and their pathogens.

## Introduction

In coordination with (or in the absence of) experimental techniques to determine genome-scale RNA secondary structure, computational methods are indispensable for identifying functional RNA structures. Such techniques are driven by RNA folding algorithms. These algorithms (such as those found in programs like RNAfold; Lorenz et al., 2011, RNAstructure; Reuter & Mathews, 2010, and UNAfold; Markham & Zuker, 2008) function under the same principle: using the Turner nearest-neighbor energy parameters (empirically derived thermodynamic parameters; Mathews et al., 1999, 2004) to predict the free energy (ΔG°) yielded during the formation of the most stable, or minimum free energy (MFE) RNA secondary structure, which is assumes that the MFE structure is, or at least closely resembles, the native secondary structure. Resulting MFE structure predictions have been shown to correctly predict ∼70% of base pairs in sequences <700 nt (Mathews, Moss & Turner, 2010); however, accuracy varies greatly by RNA.

Experimental results can be used to improve predictions of secondary structure and advances in high throughput sequencing (HTS) have facilitated the large-scale analyses of RNA structure. These techniques (such as Structure-Seq; Ding et al., 2015; Ritchey et al., 2017 and selective 2′-hydroxyl acylation analyzed by primer extension (SHAPE); Mortimer et al., 2012; Wilkinson, Merino & Weeks, 2006) are based on the use of cell-permeable small molecules, which react with nucleotides in a structure sensitive way. Modifications are detected using HTS readout of the Structure-Seq or SHAPE probing results, and can be incorporated directly into folding algorithms as constraints (Deigan et al., 2009; Washietl et al., 2012; Zarringhalam et al., 2012).

Even the most accurately predicted RNA structure, however, is incapable of suggesting whether a structure may be functional. It was observed, though, that functional noncoding (nc) RNAs had lower (more stable) MFE values than random sequences with the same nucleotide content; since the sequence of functional RNA structures are *ordered* to form a specific structure, shuffling the sequence disrupts the evolved order/structure and results in a more positive (less stable) MFE value (Babak, Blencowe & Hughes, 2007; Clote et al., 2005). This property of functional RNAs can be exploited for predictive purposes (Lim & Brown, 2017; Washietl, 2007), and is the premise behind the thermodynamic *z*-score. The thermodynamic *z*-score compares the MFE of a native sequence (MFE_native_) to the average of multiple shuffled versions (MFE_random_) and normalizes by the standard deviation (σ) of all MFE values (Eq. (1) as adapted from Clote et al. (2005)).

(1) $$ {z-{\rm{score}} = \displaystyle{{{\rm{MF}}{{\rm{E}}_{{\rm{native}}}}-\overline {{\rm{MF}}{{\rm{E}}_{{\rm{random}}}}} } \over {\rm{\sigma }}}} $$

Negative *z*-scores then, indicate that the MFE_native_ is more negative (more stable) than RNAs with the same length/nucleotide content would typically generate: for example, a *z*-score of −1 indicates the MFE_native_ is one standard deviation more stable, a *z*-score of −2 indicates the MFE_native_ is two standard deviations more stable, etc.

The *z*-score is at the heart of several functional RNA prediction approaches, including the popular program RNAz (Gruber et al., 2007, 2010; Washietl, Hofacker & Stadler, 2005b), which has been used to identify functional RNAs embedded within the human (Washietl et al., 2005a) and mouse (Thiel et al., 2018) genomes, as well as the Epstein–Barr virus (Moss & Steitz, 2013) and influenza (Moss, Priore & Turner, 2011) genomes. To span large sequences (e.g., genomes) a scanning window approach is used, where user-defined step and window sizes determine which nucleotides are analyzed: for example, defaults for RNAz window and step size are 120 and 40 nt, respectively. The justifications for small window sizes, optimally between 100 and 150 nt (Lange et al., 2012), are practical (prediction accuracy is higher for smaller RNAs), theoretical (due to the kinetics of folding, local motifs are favored) and algorithmic (RNAz, e.g., is trained on small ncRNA datasets). Another feature of many functional RNA prediction methods is the simultaneous consideration of homology in prediction: for example, align-and-fold approaches (Washietl, Hofacker & Stadler, 2005b) or fold-and-align approaches (Fu et al., 2015). Incorporating homology data can improve prediction accuracy and reduce false-positives; however, these methods are sensitive to alignment quality and sequence composition, evolutionary distance, and variation.

The ScanFold approach presented here is similar to others, in its reliance on the *z*-score, but focuses on single RNA sequences (vs. alignments) and divides the prediction process into a scanning step, a model building step, and an analysis step—where homology data or experimental results can be considered. For example, this process was previously used to map out the RNA structural landscape of the XIST long ncRNA (Fang et al., 2015). Here, as in other scanning window approaches, the challenge was to determine regions of interest for the structure modeling and analysis steps. For the study of XIST, a window *z*-score cutoff was used to define regions by overlapping low *z*-score windows. The cutoff was selected to best capture known elements, however, in many cases this may not be possible. This highlights a key drawback of scanning window approaches: individual windows are arbitrarily bounded sequence fragments while functional RNA structures are not (Will et al., 2012), which makes defining the extent of motifs a challenge.

An early approach for overcoming the problems arising from artificially bounded windows was implemented in RNAplfold (Bernhart, Hofacker & Stadler, 2006). Here, the local base pairing probabilities from multiple overlapping windows are used to generate an *average* base pairing probability for each base pair predicted throughout the scan. In this way, regions with high base pairing probability (locally stable structures) can be quickly deduced. We take a different approach in this study: here, to define the extent of local motifs, we generate *z*-score weighted consensus structures to deduce those pairs most likely to be functional.

**Figure 1:**
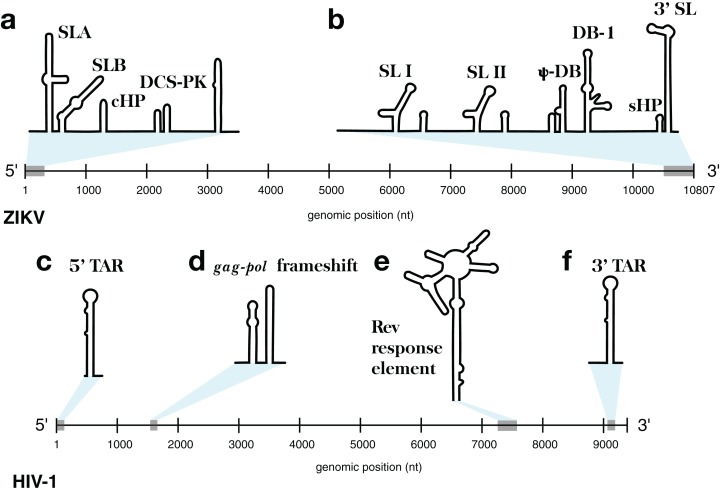
Models of the known functional RNA structural motifs found in the 5′ and 3′ end regions of the ZIKV genome and throughout the genome of HIV-1. (A) Structure model of the 5′ UTR region as shown in Ye et al. (2016). (B) Structure model of the 3′ UTR region as shown in Goertz et al. (2017). The four main RNA structural motifs of HIV-1 described in Watts et al. (2009) are shown as well: (C) the 5′TAR element; (D) the *gag-pol* frameshift element; (E) the RRE; and (F) the 3′TAR element. The relative genomic location of all structures is shown on a number line the length of its respective genome of origin.

This approach was implemented in the program ScanFold-Fold and was used to analyze the Zika virus (ZIKV) (Atieh et al., 2016) and HIV-1 (Watts et al., 2009) (+)RNA genomes. These genomes were selected for their small sizes and the known importance of RNA structure in their functions. At either end of the ZIKV genome are untranslated regions (UTRs), a short (107 nt) 5′ UTR and a longer (465 nt) 3′ UTR, which form conserved RNA structures with important functions (Goertz et al., 2017). The 5′ UTR, plus a stretch of the downstream coding region, contains several functional structured domains (Fig. 1A). The 5′ UTR has a Y-shaped stem-loop A motif, which acts as the promoter of viral genomic (v)RNA replication (Filomatori et al., 2006; Lodeiro, Filomatori & Gamarnik, 2009; Thurner et al., 2004). Directly downstream is stem-loop B, which facilitates RNA interactions with the 3′ end (Alvarez et al., 2005). The cHP domain, which overlaps the capsid protein coding region, is required for efficient vRNA synthesis; additionally, cHP enhances start codon selection (Ye et al., 2016). The DCS-PK domain enhances vRNA replication by promoting vRNA circularization (Liu et al., 2013). The 3′ UTR contains six recognized structured motifs (Goertz et al., 2017) (Fig. 1B). From the 3′ end, it contains a large stem-loop structure (3′ SL), which is required for viral replication and is highly conserved throughout flavivirus genomes (Davis et al., 2013; Elghonemy, Davis & Brinton, 2005; Villordo et al., 2016; Yu & Markoff, 2005; Zeng, Falgout & Markoff, 1998). Directly upstream is a short, well conserved hairpin (sHP), thought to be involved in genome circularization (Villordo, Alvarez & Gamarnik, 2010). Upstream of this are two structures (DB-1 and Ψ-DB), which have been shown to be conserved and duplicated, though their specific functions remains unknown (Villordo et al., 2016). The two remaining structures, SLI and SLII, which are resistant to host XRN1 exonucleases (Donald et al., 2016; Goertz et al., 2016; Pijlman et al., 2008), lead to an abundance of a highly structured ncRNA: the subgenomic flavivirus (sf)RNA, which is proposed to play roles in inhibition of the RIG-I host antiviral response (Akiyama et al., 2016; Chapman et al., 2014; Kieft, Rabe & Chapman, 2015).

The HIV-1 RNA genome, whose secondary structure has been extensively characterized (Watts et al., 2009; Wilkinson et al., 2008), contains four structured RNA elements with known functions. On either end of the genome, in the UTRs, are trans-activation response (TAR) elements (stem-loops named the 5′TAR and 3′TAR, respectively; Figs. 1C and 1F) which are involved in viral replication (Das, Klaver & Berkhout, 1998); the 5′TAR has been shown to bind the viral Tat protein during transcriptional activation (Wimmer et al., 1999) and is processed into two micro RNAs (Ouellet et al., 2008). Within the coding region of the genome are structural elements as well: the *gag-pol* frameshift element (Fig. 1D), a stem-loop structure which alters the ribosomal reading frame to allow for proper translation of the *gag* and *pol* viral proteins (which are present on overlapping reading frames), and the Rev response element (RRE), a long stem-loop structure with five terminal stem-loops (Fig. 1E), which binds viral Rev protein and allows viral mRNA to be exported from the nucleus.

Our results are compared to previously described structure models from both ZIKV and HIV-1, and tested vs. available biochemical structure probing datasets. We performed multiple benchmarking analyses of ScanFold’s ability to detect structures in the particularly well-characterized HIV-1 genome, and determined how parameters such as window size and shuffling technique affect results.

## Methods

### Data sets

The analyzed ZIKV genome was sequenced from the outbreak-lineage-derived reverse genetics system (Atieh et al., 2016) (NCBI accession KJ776791.2), and was selected to facilitate additional experimentation to better understand RNA structures’ roles in ZIKV. The sequence for HIV-1 was from the genome chemically probed by Watts et al. (2009). SHAPE reactivity profiles for ZIKV were taken from extended data 6 in Huber et al. (2018) and for HIV-1 from supplementary dataset 2 in Watts et al. (2009).

### ScanFold-Scan

The preliminary scanning window analysis for ZIKV and HIV-1 was performed by the ScanFold-Scan program (https://github.com/moss-lab/ScanFold). In this process, each window sequence is folded via RNAfold (Lorenz et al., 2011) to calculate its native MFE and associated base pairing structure at 37 °C. Each sequence is then shuffled to produce, in this case, 50 random sequences. Two different shuffling techniques were used to generate random sequences: (1) mononucleotide shuffling, which generates a random sequence with the same mononucleotide content as the native sequence and (2) Clote’s implementation of the (Altschul & Erickson, 1985) shuffling algorithm (http://clavius.bc.edu/clotelab/RNAdinucleotideShuffle/ShuffleCodeParts/altschulEriksonDinuclShuffle.txt), which generates a shuffled sequence that maintains the mononucleotide and dinucleotide content of the native sequence. Each of the 50 randomized sequences is then folded to calculate an average MFE_random_ value for use in the calculation of the thermodynamic *z*-score (see Introduction; Eq. (1)). Other metrics are calculated as well: for example, those derived from RNAfold’s use of the partition function (McCaskill, 1990) (specifically, the ensemble diversity (ED), centroid structure, and frequency of MFE, which are metrics derived from the partition function to describe the nature of an MFE’s structural ensemble), as well as a *z*-score stability ratio (calculated as the number of shuffled random MFEs which were more stable than native; referred to as the *p*-value), that can be useful as a quality control in downstream analyses. All of the aforementioned metrics are compared and described in detail in Freyhult, Gardner & Moulton (2005).

### ScanFold-Fold

The ScanFold-Fold program analyzes the output of a scanning window analysis, focusing on MFE structures and their *z*-scores. The algorithm first reads the sequence and MFE structure from every window, generating a comprehensive list of all primary sequence nucleotides (*i*), the number of windows each *i* appears in (*W_i_*), a list of all nucleotides each *i* base pairs with (*j*), and the number of windows each base pair arrangement (*i*−*j*) appears in (*W*_*i*−*j*_). For each *i*−*j*, the calculated metrics from all occurrences of the *i*−*j* are recorded and summed (e.g., for the *z*-score metric, this sum is referred to as *Z*_sum_). Next, the average MFE, ED, and *z*-score for each *i*−*j* arrangement are calculated as the sum of each metric’s value divided by *W*_*i*−*j*_; an example of this calculation is shown for the average thermodynamic *z*-score (*Z*_avg_) in Eq. (2).

(2) $${{Z_{{\rm{avg}}}} = \displaystyle{{{Z_{{\rm{sum}}}}} \over {{W_{i-j}}}}} $$

As well as average metrics observed for each *i*−*j* arrangement, a coverage-normalized *z*-score (*Z*_norm_; Eq. (3)) is calculated as *Z*_sum_ divided by the total number of windows covering *i* (*W_i_*).

(3) $${{Z_{{\rm{norm}}}} = \displaystyle{{{Z_{{\rm{sum}}}}} \over {{W_i}}}} $$

This coverage-normalized *z*-score (as opposed to *Z*_avg_) gives more weight to *i*−*j* arrangements which *consistently* appear in low *z*-score windows and provides a normalized metric for comparison of regions with lower window coverage (near the ends, where *i*’s are covered by only a few windows). This initial processing is output into a log file (an example portion of which is shown for *i*-1099 of ZIKV in Table 1).

**Table 1:** All *i*−*j* arrangements predicted for *i*-1099 of the ZIKV genome and their cumulative metrics.

*i*	*j*	nt	W_*i*−*j*_	MFE_avg_	*Z*_avg_	ED_avg_	*Z*_sum_	*Z*_norm_
1099	1099	U	7	−30.63	−0.16	31.91	−1.09	−0.01
1099	1095	A	9	−28.82	0.37	36.79	3.34	0.03
1099	1032	A	24	−29	0.28	30.65	6.74	0.06
1099	1015	A	4	−27.75	0.7	37.61	2.8	0.02
1099	1106	A	3	−30.53	0.08	21.54	0.23	0
1099	1140	G	7	−27.79	0.68	33.15	4.79	0.04
1099	1137	G	5	−29.06	0.22	34.73	1.09	0.01
1099	1087	A	5	−30.48	−0.44	34.69	−2.21	−0.02
1099	1042	A	1	−31.6	−0.42	34.99	−0.42	0
1099	1122	A	13	−32.87	−1.14	20.31	−14.83	−0.12
1099	1055	A	1	−34.7	−1.45	27.95	−1.45	−0.01
1099	1082	A	11	−33.64	−1.71	27.5	−18.82	−0.16
1099	1104	A	16	−33.33	−1.68	23.25	−26.82	−0.22
1099	1177	A	9	−31.43	−0.89	21.01	−8.03	−0.07
1099	1080	G	2	−29.6	−0.6	25.53	−1.2	−0.01
1099	1189	A	2	−28.3	−0.41	28.36	−0.82	−0.01
1099	1182	G	1	−31.6	−0.67	25.74	−0.67	−0.01

For each *i* in the sequence, a single *i*−*j* arrangement is selected to represent the most favorable arrangement; here the “most favorable” arrangement is considered to be the one with the lowest *Z*_norm_. Selection based on *Z*_norm_ results in a list of the most favorable *i*−*j* arrangements for every *i* of the input sequence. Importantly, the ScanFold-Fold algorithm must consider upstream and downstream base pairing competition when selecting the “best” *i*−*j* arrangement; it is possible that different *i*’s will compete for the same *j*, which would result in the generation of unrealistic models depicting single nucleotides paired with multiple partners.

In cases of competition, such as shown in Table 2, where all three *i*’s compete for the same *j*-1104, only one *i* can be selected to partner with *j*-1104. Here, the lowest *Z*_norm_ among the set of competing *i*−*j* arrangements was observed for the unpaired arrangement (*i*-1104 and *j*-1104); therefore, *j*-1104 is awarded to *i*-1104 and an assumption is made that *i*-1099 and *i*-1078 do not pair with *j*-1104; for the sake of the consensus model, they will remain unpaired (see *i*-1099 in Table 3 where the final partner for *i* is shown to be *i*-1104 and *j*-1104; the * indicates that the winning *j* for *i*-1099 had a more favorable arrangement which is reported in its place).

**Table 2:** Example of most favorable *i*−*j* arrangements which compete for the same *j* nucleotide.

*i*	*j*	nt	*W*_*i*−*j*_	*Z*_avg_	*Z*_sum_	*Z*_norm_
1104	1104	A	48	−0.57	−27.5	−0.23
1099	1104	A	16	−1.68	−26.82	−0.22
1078	1104	A	11	−1.71	−18.82	−0.16

**Table 3:** List of final *i*−*j* arrangements and their average metrics for nucleotides 1099–1108 of the ZIKV genome.

*i*	*i*	*j*	MFE_avg_	*Z*_avg_	ED_avg_
1099*	1104	1104	−31.31	−0.57	26.24
1100	1100	1100	−32.9	−1.27	23.94
1101	1101	1101	−32.1	−1	25.77
1102	1102	1102	−31.48	−0.75	27.21
1103	1103	1103	−31.67	−0.82	25.13
1104	1104	1104	−31.31	−0.57	26.24
1105	1105	1105	−31.26	−0.55	26.76
1106	1106	1106	−31.13	−0.57	26.92
1107	1107	1107	−30.99	−0.47	27.4
1108:	1108	1108	−31.31	−0.65	27.11

These results are printed to a file as well; an example portion of which is shown in Table 3, for ZIKV nt’s *i*-1099 to *i*-1108). Ultimately, this selection process allows for the generation of connectivity table (CT) files which span the entire genome.

### Filtering

**Figure 2:**
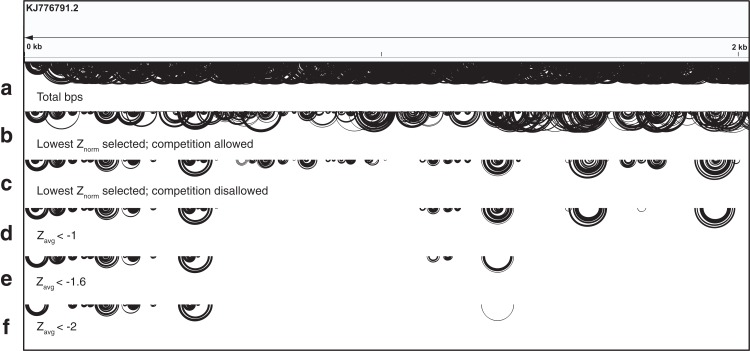
Depiction of the ScanFold-Fold processing of scanning window results. This image depicts the base pairs identified on the first 2,000 nts of the ZIKV genome (accession KJ776791.2) through each step of ScanFold-Fold processing, as base pairing tracks (Busan & Weeks, 2017) on IGV (Thorvaldsdottir, Robinson & Mesirov, 2013). (A) The first track shows the totality of base pairs predicted throughout the ScanFold-Scan process. (B) The second track depicts the base pairs which remain after ScanFold-Fold selects the most favorable base pair (according to the lowest *Z*_norm_; see Methods Eq. (3)) per *i* nucleotide of the sequence; competition is allowed, that is, multiple partners are permitted to pair with the same nucleotides. (C) The third track shows the base pairs which remain after prohibiting multiple pairing partners per nucleotide; that is, competition is disallowed whereby only a single pairing partner is allowed per *i* or *j* nucleotide. This track is equivalent to the “no filter” results from ScanFold-Fold. The base pairs from this track are then subjected to filtering based on their *Z*_avg_ (see Methods Eq. (2)). The final tracks depict which base pairs from the results above possessed *Z*_avg_ scores less than (D) −1 (E) −1.6 (one standard deviation below the mean *z*-score) and (F) −2.

Since the primary interest for this analysis is to reveal potentially functional structures, a filtering process is employed to hone in on base pairs common to analysis windows with highly negative *z*-scores. For this filtering process, *Z*_avg_ for each *i*−*j* arrangement is considered; only *i*−*j* arrangements with a *Z*_avg_ below a filter value are written to a CT file. An example of this filtering process is shown in Fig. 2 for the first 2,000 nt of ZIKV (full genome in Fig. S1). By default, the ScanFold-Fold program will write multiple CT files, each using a different filter value: −2, −1, 10 (no filter) are used by default, and a user-defined value is also allowed. The user-defined filter value is provided because the definition of a “significantly” negative *z*-score can vary (see “Comparison of shuffling techniques” in Results) and users may want to select different values.

### Alignment

A total of 37 ZIKV genomes (curated in the ZikaVR database; Gupta et al., 2016) were aligned to the scanned ZIKV genome (accession KJ776791.2) using the MAFFT web server (Katoh, Rozewicki & Yamada, 2017; Kuraku et al., 2013) with default settings. Aligned sequences were compared to ScanFold-Fold predicted base pairs (with *Z*_avg_ < −1) to tabulate the types of base pairs that are found throughout the alignment; nucleotides with mutations which maintained a ScanFold-Fold predicted base pair are noted in figures as “structure-preserving.”

## Results

### ScanFold-Fold predicted motifs in the ZIKV genome

**Figure 3:**
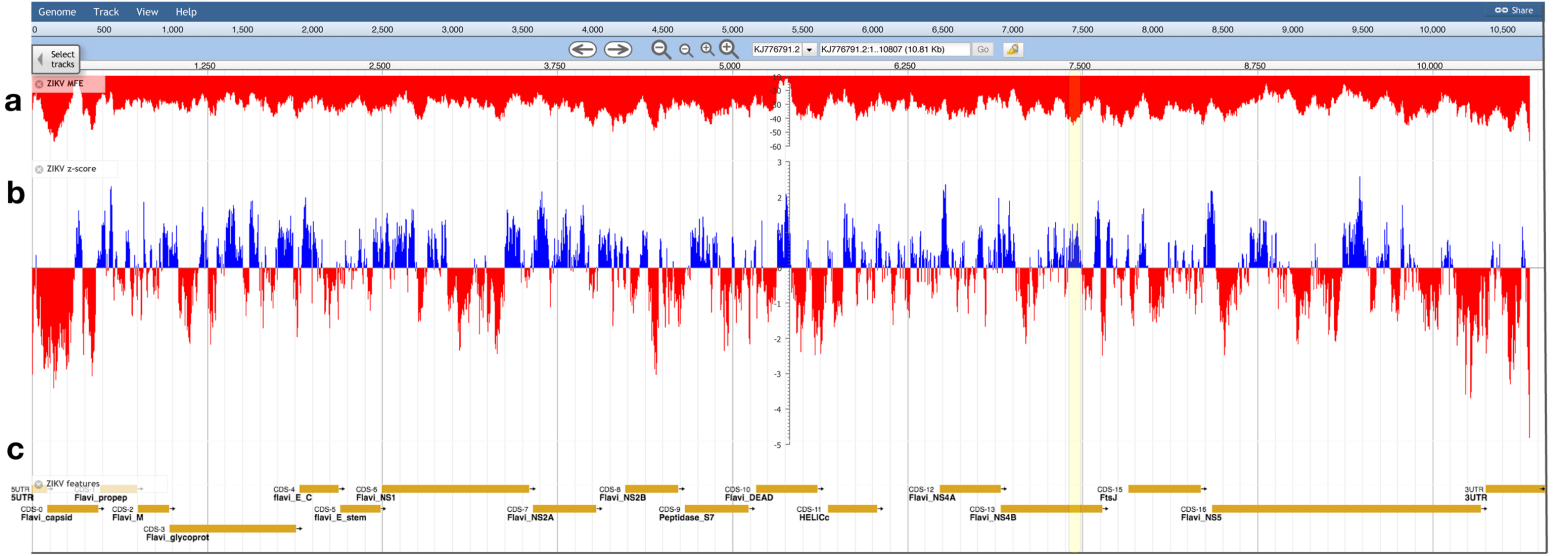
Bioinformatics scans of the ZIKV genome. (A) The predicted minimum folding free energy (MFE) and (B) *z*-score for all RNA window segments; red and blue colors indicate negative and positive values, respectively. Each bar is set at the first nt of the analysis window. The most striking region where a low MFE ΔG did not correlate with a negative *z*-score is highlighted in yellow. (C) Genome feature annotations are shown; the polyprotein region has been broken down for visualization of individual coding sequence regions. All data was visualized, archived, and is available for browsing/download on the RNAStructuromeDB https://structurome.bb.iastate.edu/.

The ZIKV genome was analyzed with ScanFold-Scan using a 120 nt window with a one nt step: resulting in 10,688 analyzed windows (Table S1). For each window, several metrics of RNA folding were predicted (described in the Results and Discussion of Andrews, Baber & Moss (2017)); two metrics of particular interest are plotted vs. the ZIKV genome in Fig. 3: the MFE (Fig. 3A) and the thermodynamic *z*-score (Fig. 3B; Eq. (1)) using a mononucleotide shuffling technique. Across the ZIKV genome are windows where low MFEs overlap low *z*-score windows, but also places where they do not (Fig. 3: highlighted in yellow). Even for a relatively small genome such as ZIKV, 3,349 windows had *z*-scores less than −1 (signifying the window MFE prediction was one standard deviation more stable than random) and 994 windows with *z*-scores less than −2. With so many windows of interest, and so many competing models, it is a challenge to identify the most functionally significant base pairs. This was the impetus behind the development of ScanFold-Fold: to identify the base pairs which were responsible for generating low *z*-score regions and that persisted across multiple overlapping analysis windows.

In total, 22,180 unique base pairs were predicted throughout all scanning windows (Fig. S1A; Table S2); some nucleotides were predicted to form base pairs with as many as 16 different partners, highlighting the challenge of finding a single model (e.g., *i*-1099; Table 1). ScanFold-Fold records the metrics from each window where the base pair appears, generating a set of cumulative metrics. For each *i*, only one base pairing partner is selected. Selecting base pairs with the lowest *Z*_norm_ (Eq. (3)) and *allowing* competition (see Materials and Methods) yielded a smaller group of 6,831 base pairs (Fig. S1B). *Disallowing* competition (see Materials and Methods), however, yields a much smaller group of 2,259 base pairs (Fig. S1C; Table S3). To focus on the most significant hits, cumulative *z*-score filters were applied to identify the base pairs which were consistently found in low *z*-score windows: *Z*_avg_ filters of −1 and −2 were used.

With a *Z*_avg_ filter of −1 and 1,114 base pairs were identified (Fig. S1D; Table S4). Consistent with the presence of structured functional motifs, many base pairs were found within the known ZIKV structured regions; a total of 194 base pairs were found within previously identified 5′ and 3′ end structured domains. ScanFold-Fold was able to identify 86 of the 114 known base pairs (Goertz et al., 2017) in the 3′ UTR (Fig. S2) and 75 of the 81 known base pairs (Ye et al., 2016) within the 5′ structured domain (Fig. S3).

**Figure 4:**
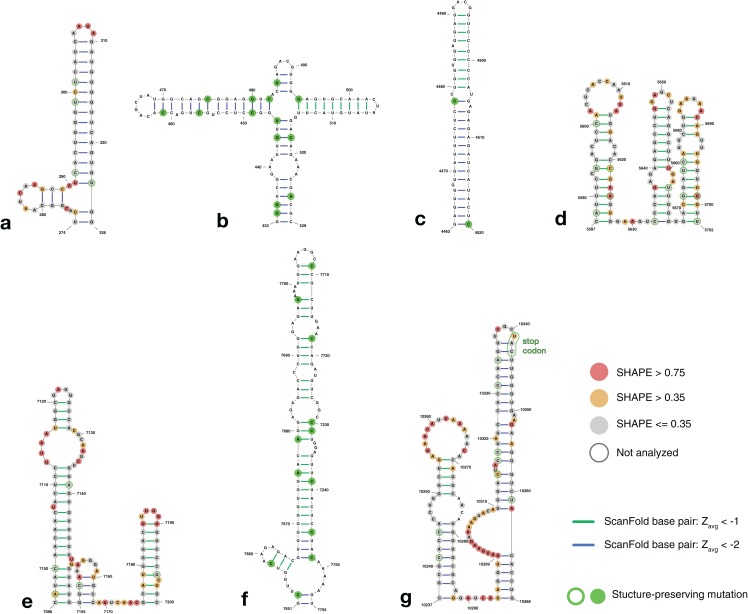
Structure models of ZIKV coding region motifs which contain ScanFold-Fold base pair hits with *Z*_avg_ < −2. Structures are shown in the order they appear throughout the ZIKV genome (KJ776791.2). (A) and (B) depict the structural models of the ScanFold-Fold predicted motifs adjacent to the previously annotated 5′ structured region, located from nt 274–326 and 433–529, respectively. (C–F) are structures that appear within the core coding region located from nt 4,463–4,520, 5,587–5,702, 7,096–7,205, and 7,651–7,755, respectively. Structure (G) is directly upstream of the 3′ structured region, located at nt 10,237–10,368, and has been annotated to show the location of the stop codon near the terminal loop (circled in green). Base pairs are colored by their *z*-score cutoff; green lines show base pairs which were predicted in the *z*-score < −1 results (Fig. S1D; Table S4) and blue lines show base pairs which were predicted in *z*-score < −2 results (Fig. S1E; Table S5). Sites with structure-preserving mutations are highlighted with green circles. All nucleotides are shown with their SHAPE reactivity scores as shown in Huber et al. (2018).

Further filtering the results to *Z*_avg_ < −2 reduced the number of base pairs to 233 (Fig. S1F; Table S5). Of these, 121 were found in known structural regions. Interestingly, regions immediately adjacent (within 240 nt, or two window lengths) to these known functional motifs also had 86 *Z*_avg_ pairs < −2 (Figs. 4A, 4B and 4G), suggesting that the regions of functional structure at either end may be larger than previously thought (full structural models of the extended 5′ and 3′ ends are shown in Fig. S4). The remaining 26 base pairs contribute to four structures found within the core coding region (Figs. 4C–4F).

To determine the structural conservation of these ScanFold-Fold identified base pairs, an alignment was performed of 37 ZIKV genomes curated in the ZikaVR database (Gupta et al., 2016); aligned sequences are reported in Table S6. The ScanFold base pairs with *Z*_avg_ < −1 were mapped to the alignment to determine the conservation of base pairing across ZIKV genomes. When mutations occurred in predicted paired regions they generally preserved base pairing: for example, ScanFold-Fold predicted base pairs were over 96% conserved (Table S7). Multiple structure-preserving mutations occur throughout novel predicted motifs (Fig. 4) as well as in previously described ZIKV structures (Fig. S4).

### ScanFold-Fold identified motifs in the HIV-1 genome

**Figure 5:**
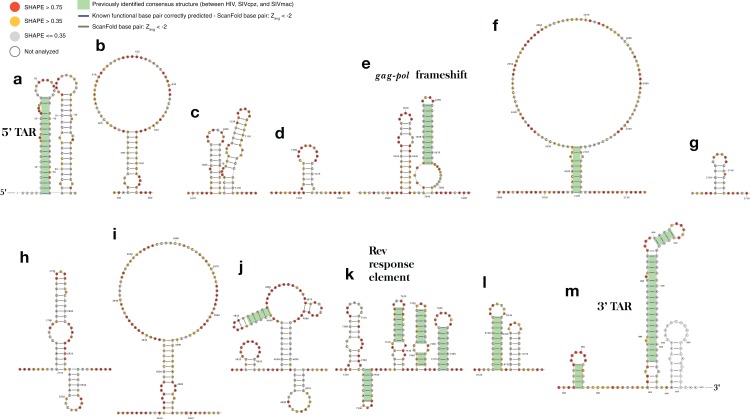
ScanFold-Fold identified base pairs in the HIV-1 genome. All base pairs shown were predicted to have a *Z*_avg_ < −2, using ScanFold-Scan with a 120 nt window length, one nt step size, and mononucleotide shuffling 50 times. All nucleotides are depicted with their SHAPE reactivity scores from Watts et al. (2009) (annotated using VARNA; Darty, Denise & Ponty, 2009 with the colors mapped on a gradient to depict a reactivity ≤0.35 as gray, a reactivity >0.75 as red and a reactivity between 0.35 and 0.75 as yellow). All base pairs which have been highlighted with a green box were identified as conserved “consensus structures” in a comparative analysis between HIV-1 and two lentiviral relatives, SIVcpz and SIVmac (Lavender, Gorelick & Weeks, 2015). All structures are labeled from (A) to (M) based on their genomic position from 5′ to 3′, with all known functional structures labeled with their names.

In order to benchmark the ScanFold pipeline, ScanFold-Fold *Z*_avg_ < −2 base pairs were compared to well-characterized, experimentally-supported models for HIV-1 RNA structural motifs. Using the same pipeline used for the ZIKV genome, 13 structured regions were identified that contained base pairs with *Z*_avg_ < −2. These regions are shown in Fig. 5. Again, all previously described RNA structural elements were identified with the ScanFold pipeline; the 5′TAR element (Fig. 5A), the *gag-pol* frameshift element (Fig. 5E), the five terminal stem loops of the RRE (Fig. 5K), and the 3′TAR element (Fig. 5M) nucleotides were all modeled to be in structures consistent with previous descriptions and in are good agreement with SHAPE reactivity (with the exception of the first hairpin in the RRE).

Interestingly, three of the remaining structures (Figs. 5F, 5J and 5L) contain the same structurally conserved base pairs as were previously identified in a comparative analysis with two primate lentiviral SIV strains (see Figs. 3 and 4 from Lavender, Gorelick & Weeks (2015)) and are also in agreement with SHAPE reactivity data. The remaining structures, while not previously described, are in good agreement with SHAPE reactivity data (with some slight discrepancies for the first hairpin of the structures in Figs. 5H and 5K).

### Comparison of shuffling techniques

**Figure 6:**
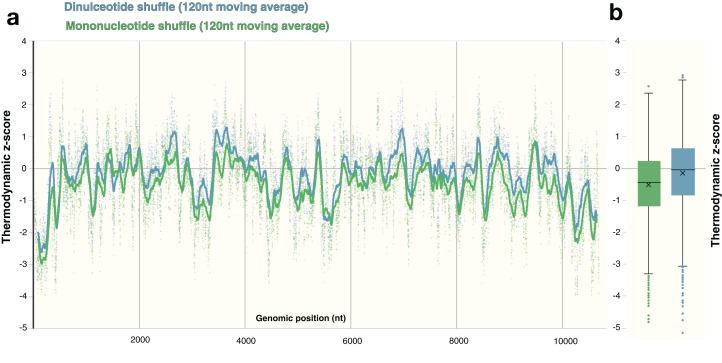
Comparison of shuffling techniques. The ZIKV genome was analyzed with ScanFold-Scan using a 120 nt window size, one nt step size, and 50 iterations of either mononucleotide or dinucleotide shuffling during the calculation of the thermodynamic *z*-score. (A) For each window, the calculated *z*-score is plotted as a point along the genome (positioned according to the starting coordinate of the respective window). To observe the general *z*-score trend present for each shuffling technique, a moving average for every 120 points is plotted as a line against genomic position as well; where green and blue coloring refers to mononucleotide and dinucleotide shuffling, respectively. (B) The same data from above is plotted here as whisker plot: center lines indicate the median; crosses indicate the mean; the box limits indicate the 75th and 25th percentiles; whiskers extend to 1.5 times the interquartile range; outliers are represented as dots.

The process of shuffling RNA can affect the *z*-score (Forsdyke, 2007). Dinucleotide shuffling preserves nearest-neighbor nucleotides (that can stack in helixes), while mono-nucleotide shuffling abolishes this pattern—potentially overestimating the magnitude of the *z*-score (Gesell & Washietl, 2008). To determine the impact of mono- vs. dinucleotide shuffling on ScanFold results (which rely primarily on the thermodynamic *z*-score), both dinucleotide and mononucleotide shuffling were performed on ZIKV and HIV-1. These two shuffling techniques were implemented during each analysis to identify the differences in the resulting *Z*_avg_ base pairs. For the ZIKV genome, the same overall *z*-score pattern (resulting in identification of similar motifs) was observed between shuffling techniques (Fig. 6A), however the mean *z*-score across the genome differed slightly: −0.55 and −0.18 for mononucleotide and dinucleotide shuffling, respectively (Fig. 6B). Dinucleotide shuffling results using a *Z*_avg_ cutoff of −2 yielded fewer base pairs (147 bps) than mononucleotide shuffling (233 bps); this is likely due to the generally more positive *z*-scores that arise from using dinucleotide shuffling. Overall, the results for the known structures in the UTR regions are the same between shuffling techniques (Fig. S5); however, in the 3′ UTR, mononucleotide shuffling detected DB-1 and Ψ-DB with a *Z*_avg_ < −2 while dinucleotide shuffling did not. The other differences between results can be seen in the coding region. Between the two techniques, eight structured regions were identified in the coding region of ZIKV with *Z*_avg_ < −2 base pairs (Figs. S5A–S5H), half of which were identified by both techniques with slight differences in the quantity and location of base pairs (Figs. S5A, S5C, S5D and S5H). Three structured regions were identified exclusively by mononucleotide shuffling (Figs. S5B, S5E and S5F) and one structured region was exclusive to dinucleotide shuffling (Fig. S5D).

**Figure 7:**
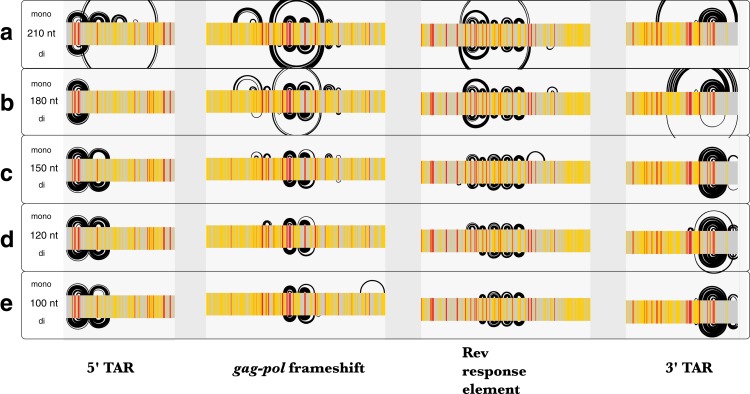
Comparison of ScanFold-Fold results for known HIV-1 structures against SHAPE reactivity data when using different shuffling and window sizes. The base pairs predicted by ScanFold-Fold (with *Z*_avg_ scores < −2) which appear near the known functional RNA structures from HIV-1 are shown here as base pairing tracks using IGV (genome scale results shown in Fig. S6). Each of these tracks depicts results when using a mononucleotide (top) or dinucleotide (bottom) shuffling technique. Each of the five tracks shows results when using a different window size: (A) 210 nt, (B) 180 nt, (C) 150 nt, (D) 120 nt (the default window size), and (E) 100 nt. SHAPE reactivity data from Watts et al. (2009) is shown as a heat map for each track, where a reactivity ≤0.35 is colored gray, a reactivity >0.75 is red and a reactivity between 0.35 and 0.75 is yellow.

Similar to Zika, the average *z*-scores for the HIV-1 genome were more positive using dinucleotide shuffling than mononucleotide shuffling (−0.15 and −0.49, respectively). Minimal differences were observed between each shuffling technique’s ability to detect known structured regions over a range of window sizes (Fig. 7). Most of the base pairs identified in these regions were identical, where the only differences are due to mononucleotide shuffling detecting more base pairs: for example, there were 18 more identified pairs in regions found using a 120 nt window (Fig. 7D). This is consistent with the more positive *z*-scores obtained using dinucleotide shuffling overall, where a less stringent *Z*_avg_ filter would likely identify more base pairs. Though there were differences between the number of base pairs identified in the regions intervening the known structural elements, identified base pairs were consistent with SHAPE reactivity data (Fig. S6).

### Comparison of results from different window sizes

Since the MFE structures predicted throughout scanning window analyses are sensitive to window size, the HIV-1 genome was analyzed using five different window sizes (100, 120 150, 180, and 210 nt) and a *Z*_avg_ filter of −2. The results obtained using each window size were compared to published SHAPE reactivity profiles from Watts et al. (2009). All window sizes using a mononucleotide shuffle correctly identified base pairs from the previously-described structural elements of HIV-1 (Fig. 7); where larger window sizes generally identified more base pairs around known functional structures (with a single exception: using a window size of 180 nt, none of the base pairs from the poly(A) stem located directly upstream of 5′TAR element (Lavender, Gorelick & Weeks, 2015) had *Z*_avg_ below −2 (Fig. 7B).

In regions with no previously-described functional structures, base pairs with *Z*_avg_ < −2 are consistent with the SHAPE reactivity profiles as well (Fig. S6), however, the location and quantity of bases pairs differs between window sizes. In general, the number of base pairs identified increased as window size increased, while prediction accuracy in known structured regions remained the same or was diminished. This suggests that a window size between 100 and 150 nt may be optimal; this is consistent with findings from a previous study that aimed to identify the optimum window size for detection of structured regulatory elements embedded in long mRNA molecules (Lange et al., 2012).

## Discussion

The ScanFold-Fold analysis of ZIKV reiterates the prominence of RNA structure in the 5′ and 3′ regions of its genome, indicates that these (previously described) structured regions could be larger, and reveals several potentially functional structures within the core coding region. This was achieved by an approach which condenses thousands of scanning window models into a list of base pairs with the highest likelihood of generating functional RNA structures; greatly reducing the dependence on subjective manual curation of results. The ScanFold-Fold algorithm was able to successfully identify known base pairs within the 5′ and 3′ regions with high positive predictive value (PPV) and sensitivity (Table S8) (Bellaousov et al., 2013; Mathews, 2004; Mathews et al., 1999). In the 5′ UTR region, ScanFold-Fold positively identified all known structures (with slight variations; Fig. S3), while the 3′ UTR model was accurate in identifying the structures of all elements, except for regions of the exonuclease resistant SLI and SLII structures (Fig. S2); likely due to the presence of complex RNA pseudoknot structures here (Akiyama et al., 2016), which can be difficult to predict computationally due to non-nested base pairing that complicates the use of recursive algorithms (Schlick & Pyle, 2017).

ScanFold-Fold identified structures directly upstream and downstream of the 5′ and 3′ UTR regions (nt 270–528 and nt 10,237–10,370, respectively) have metrics that are equally as favorable as those in the known structural regions. These structures (Figs. 4A, 4B and 4G) have unusually stable thermodynamic stability (compared to random), are well conserved throughout ZIKV genomes (Table S7), and the structures in Figs. 4A and 4G are supported by SHAPE reactivity data (reactivity data for the structure in Fig. 4B was not reported). Their close proximity to functional UTR regions (Fig. S4) suggests that they may play important roles in conjunction with these other elements: for example, in genome replication, processing, etc. The structures predicted in the core coding region (Figs. 4C–4F), also have metrics that indicate they may have evolved to form functional conformations, are conserved, and SHAPE reactivity data (where available) agrees with these models (Figs. 4D and 4E). There are numerous potential functions for these core region RNA structural motifs: for example, serving as sites of post-transcriptional modifications (Coutard et al., 2017; Dong et al., 2014; Gokhale et al., 2016; Lichinchi et al., 2016), facilitating packaging of the genome (Stockley et al., 2013), acting as localization signals (Pratt & Mowry, 2013), modulating the rate of translation to affect protein folding (Khrustalev et al., 2017), or to affect transcript (genome) stability (Wu & Brewer, 2012).

Known and novel ZIKV motifs predicted by ScanFold-Fold are corroborated by a recent study of ZIKV combining biochemical structure probing with comparative analysis (Huber et al., 2018; Extended data 6). This highlights the robustness of ScanFold-Fold predicted models and the program’s ability to rapidly deduce likely functional motifs. Similarly, our benchmarking of ScanFold-Fold using HIV-1 data also showed good correlations with predictions and experimental data, and demonstrate its ability to independently identify functional (e.g., named) RNA motifs. Interestingly, in addition to named HIV-1 motifs, several additional structures were identified (Figs. 5F, 5J and 5L) which contain the same structurally conserved base pairs identified in a comparative analysis of HIV-1 with two primate SIV strains (Lavender, Gorelick & Weeks, 2015). No functions are currently proposed for these structures, however, their folding metrics are highly suggestive of their importance.

The map of the RNA structural landscape of the ZIKV genome and the reanalysis of HIV-1 presented in this report serves as a guide for future analyses. The functional importance of novel ScanFold-Fold identified motifs can be tested in virio by designing mutations to disrupt/compensate structure (e.g., using a tool such as RNA2DMut; Moss, 2018)—while maintaining (or minimally disrupting) amino acid coding (and codon use)—then introducing them into the genome: for example, via ZIKV or HIV-1 genetics systems (Atieh et al., 2016; Smyth et al., 2014) to assess effects on viability, infectivity, and replication. A similar strategy was previously used to test RNA structural motifs predicted to occur in influenza A virus (Jiang et al., 2016). Furthermore, the presence of conserved base pairing within coding regions would be expected to impact their evolution (to maintain both functional protein and RNA structure); thus, these data can also potentially be helpful in understanding constraints placed on the evolution of these viruses. This is particularly significant to understanding the evolution and outbreak of pathogenic ZIKV strains.

### Considerations

The underlying folding algorithm used in ScanFold-Scan, RNAfold (version 2.3.3), has been extensively benchmarked vs. experimental data (Lorenz et al., 2011), and is one of the top performing single-sequence, energy-based folding algorithms available (Puton et al., 2014). Despite the similarity in prediction accuracies between other top performing folding algorithms (such as RNAstructure; Reuter & Mathews, 2010 and UNAfold; Markham & Zuker, 2008), differences between their MFE structure predictions still arise due to the different ways the Turner energy parameters are implemented. It should be noted that output from any RNA folding algorithm (Puton et al., 2014), when properly formatted, can be considered in the ScanFold-Fold process. This could address specific inaccuracies or limitations of any particular approach. Indeed, one could combine results from multiple predictions approaches in ScanFold-Fold to get consensus motifs. Likewise, another way to address algorithm limitations is to incorporate data from biochemical structural analyses (Deigan et al., 2009; Washietl et al., 2012; Zarringhalam et al., 2012). Although, unconstrained ScanFold-Fold results were consistent with SHAPE data from ZIKV and HIV-1, these data could have also been included as constraints in the ScanFold-Scan window analyses: for example, by constraining reactive bases in each window.

A limitation of the ScanFold procedure is that base pairing beyond the window size used cannot be predicted. For example, functional long-range RNA–RNA interactions (LRIs) have been identified within genomes of positive-strand RNA viruses such as ZIKV (Huber et al., 2018). These interactions span thousands of nucleotides (much greater in distance than the typical scanning analysis window) and play functional roles in viral transcription, translation, and replication (Nicholson & White, 2014). Because they span such large distances, scanning window approaches are unable to explicitly predict LRIs; however, by deducing local regions with high propensity of folding, these no longer need to be considered when trying to deduce LRIs.

The ScanFold-Scan approach presented here was developed as a single sequence alternative to approaches for functional RNA motif discovery. It differs from alignment-based methods such as RNAz and locARNA by dividing the analysis steps to allow phylogenetic comparisons to be run after folding. In this way, ScanFold-Scan and ScanFold-Fold can be used to detect both conserved and nonconserved elements, which may be significant for recently-evolved viral strains, for example. It should be noted, however, that output from alignment-based approaches are also compatible with ScanFold-Fold and can readily be used to detect conserved elements from alignments.

## Conclusions

In conclusion, this report presents a bioinformatics scan of the ZIKV and HIV-1 genomes and a novel analysis pipeline/method for functional RNA motif discovery that (1) recapitulates known functional motifs in both viruses, (2) suggests that regions of RNA structure in ZIKV may be larger than previously reported, (3) finds novel motifs that may be functionally important, and (4) provides a road-map for testing the functions of RNA structure in the biology of both ZIKV and HIV: for example, by disrupting structure via mutations to viral genomes.

## Supplemental Information

10.7717/peerj.6136/supp-1 Supplemental Information 1 Fig. S1. Depiction of the ScanFold-Fold processing of scanning window results for the full ZIKV genome. This image depicts the base pairs identified on the full length of the ZIKV genome (accession KJ776791.2) through each step of ScanFold-Fold processing, as base pairing tracks (Busan & Weeks, 2017) on IGV (Thorvaldsdottir, Robinson & Mesirov, 2013). (a) The first track shows the totality of base pairs predicted throughout the ScanFold-Scan process. (b) The second track depicts the base pairs which remain after ScanFold-Fold selects the most favorable base pair (according to the lowest Z_norm_; see Methods Eq. 3) per *i* nucleotide of the sequence; competition is allowed, whereby multiple partners are permitted to pair with the same nucleotides. (c) The third track shows the base pairs which remain after prohibiting multiple pairing partners per nucleotide; competition is disallowed, i.e. for nucleotides predicted to have more than one most favorable partner, only a single pairing partner is allowed, selected as the one with the lowest Z_norm_. This track is equivalent to the “no filter” results from ScanFold-Fold. The base pairs from this track are then subjected to filtering based on their Z_avg_ (see Methods Eq. 2). The final tracks depict which base pairs from the results above, generated Z_avg_ scores less than (d) −1 (e) −1.6 (one standard deviation below the mean z-score) and (f) −2.

10.7717/peerj.6136/supp-2 Supplemental Information 2 Fig. S2. Comparative arc diagram depicting the previously described 5’ RNA structural motifs vs. the ScanFold predicted base pairs. (a) Arc diagram of the 5′ end region as predicted via ScanFold-Fold; base pairs are colored by their z-score cutoff where blue lines depict base pairs which were predicted in the z-score < −2 results (Table S7) and green lines refer to base pairs which were predicted in the z-score < −1 results (Table S6). (b) Arc diagram of the accepted secondary structure model for the 5′ end of ZIKV as shown in (Ye et al., 2016) and mapped to the KJ776791.2 sequence.

10.7717/peerj.6136/supp-3 Supplemental Information 3 Fig. S3. Comparative arc diagram depicting known RNA structural motifs vs. ScanFold-Fold predicted base pairs. (a) Arc diagram of the 3′ end region as predicted via ScanFold; base pairs are colored by their z-score cutoff where blue lines depict base pairs which were predicted in the z-score < −2 results (Table S7), green lines refer to base pairs which were predicted in the z-score < −1 results (Table S6), and yellow lines were predicted in the no filter results (Table S5). (b) Arc diagram of the accepted secondary structure model for the 3′ end of ZIKV as shown in (Goertz et al., 2017) mapped to the KJ776791.2 sequence. The start codon nucleotide locations have been highlighted with a light blue bar.

10.7717/peerj.6136/supp-4 Supplemental Information 4 Fig. S4. Secondary structure model depicting the ScanFold proposed structures within and directly adjacent to known 5′ and 3′ structured regions. Base pairs are colored by their z-score cutoff: blue lines depict base pairs which were predicted in the z-score < −2 results (Table S7), green lines refer to base pairs which were predicted in the z-score < −1 results (Table S6), and yellow lines were predicted in the no filter results (Table S5). The start and stop codon nucleotides have been circled and labeled in blue and green respectively. Nucleotides which established ScanFold base pair preserving mutations within the alignment are highlighted with filled green circles. The SHAPE reactivity scores available from dataset 6 of (Huber et al., 2018) have been mapped onto nucleotides (where data is available).

10.7717/peerj.6136/supp-5 Supplemental Information 5 Fig. S5. Comparison of ScanFold-Fold results for the ZIKV genome using different shuffling techniques. The entirety of base pairs predicted throughout the genome of ZIKV have been plotted as RNA base pairing tracks using IGV. Three Z_avg_ filter values were used and plotted as separate tracks (labeled as such). Each of these tracks depicts results when using a mononucleotide (top) or dinucleotide (bottom) shuffling technique. The known structured regions on the 5′ and 3′ end have been highlighted in blue. The novel structures predicted (with Z_avg_ scores < −2) in the core coding region have been highlighted in yellow and labeled (a) to (h) based on their genomic location.

10.7717/peerj.6136/supp-6 Supplemental Information 6 Fig. S6. Comparison of ScanFold-Fold results for the HIV-1 genome when using different shuffling and window sizes, alongside SHAPE reactivity data. Here, the complete set of ScanFold-Fold Z_avg_ < −2 base pairs generated across the HIV-1 genome are depicted as RNA base pairing tracks (Busan & Weeks, 2017) on IGV (Thorvaldsdottir, Robinson & Mesirov, 2013). Each of these tracks depicts results when using either a mononucleotide (top) or dinucleotide (bottom) shuffling technique to calculate the z-score. Each of the five tracks shows results when using a different window size: (a) 210 nt (b) 180 nt (c) 150 nt (d) 120 nt (the default window size) and (e) 100 nt. SHAPE reactivity data from (Watts et al., 2009) is shown as a heat map for each track where a reactivity < = 0.35 is colored grey, a reactivity > 0.75 is red and a reactivity between 0.35 and 0.75 is yellow.

10.7717/peerj.6136/supp-7 Supplemental Information 7 Table S1. Results of the scanning window analysis of the ZIKV genome (NCBI accession KJ776791.2) as output from the ScanFold-Scan program. Each row contains the data calculated for each window. Columns A and B are the starting (i) and ending (j) coordinates of the window fragment. Column C is the temperature used for all RNAFold calculations. Column D-H refer to the ΔG_native_, thermodynamic z-score, stability ratio p-value, ensemble diversity, and f requency-of-MFE (fMFE) values respectively (detailed descriptions of all metrics can be found at the RNAStructuromeDB https://structurome.bb.iastate.edu or the corresponding manuscript (Andrews, Baber & Moss 2017)). Column I contains the sequence of the window; the ΔG_native_ and centroid structure of this sequence are shown in Column J and K. Column L-O report nucleotide counts for the window sequence.

10.7717/peerj.6136/supp-8 Supplemental Information 8 Table S2. ScanFold log file produced during the ScanFold-Fold portion of the program. The log file is separated into two portions. The first half (row 1 to 87,448) contains a table for each nucleotide in the sequence. These tables contain the cumulative base pairing information for that nucleotide as predicted throughout the scan. Column A refers to the *i*-nucleotide of the sequence. Column B refers to the coordinate of the *j* base pair. The total number of windows the *i-j* pair appears, as well as the total number of windows the *i*-nucleotide appears are reported in column D. The average window minimum free energy, z-score, and ensemble diversity of each *i-j* pair are reported in columns E-G respectively. Column H reports the *sum* of z-scores for each *i-j* pair, which is used to calculate the coverage-normalized z-score (calculated as the sum of z-score over total windows in which *i*-nucleotide appeared) as reported in Column I. Column J reports a summary of the base pairs predicted for each *i*-nucleotide. The second half of the log file, starting at row 87,449, is a list of the most favorable *i-j* pairs (column B and C) associated with the *i*-nucleotide listed in column A. In places where this nucleotide competed with other *i*-nucleotides for the same *j*-nucleotide, the “winning” *i-j* pair is reported and denoted with an asterisk (in some cases the winning *i-j* pair does not contain the original *i*-nucleotide or may be unpaired). Columns D, E, and F, contain the average window minimum free energy, z-score and ensemble diversity for the corresponding *i-j* pair.

10.7717/peerj.6136/supp-9 Supplemental Information 9 Table S3. Results of 37 ZIKV genomes curated in the ZikaVR database (Gupta et al., 2016) aligned to KJ776791.2. Genomes were aligned using the MAFFT web server (Katoh, Rozewicki & Yamada, 2017; Kuraku et al., 2013) with default settings. Headings for each result contain the NCBI accession numbers and name of the aligned sequence name.

10.7717/peerj.6136/supp-10 Supplemental Information 10 Table S4. Base pair counts tabulating the number and type of base pair which appears in the ScanFold < −1 predicted structure when compared to 37 aligned ZIKV genome. A total of 37 ZIKV genomes were aligned to KJ776791.2 using the MAFFT web server (Katoh, Rozewicki & Yamada, 2017; Kuraku et al., 2013) using default settings. Aligned sequences were compared to ScanFold-Fold predicted base pairs (with z-score < −1) to tabulate the types of base pairs which are found throughout the alignment (Table S3). Column S reports the percent of canonical base pairs which were found to be allowed throughout the alignment for that base pair and column T reports the different number of canonical base pair types.

10.7717/peerj.6136/supp-11 Supplemental Information 11 Table S5. CT file of the default no-filter results output from ScanFold-Fold.

10.7717/peerj.6136/supp-12 Supplemental Information 12 Table S6. CT file of the default z-score < −1 results output from ScanFold-Fold.

10.7717/peerj.6136/supp-13 Supplemental Information 13 Table S7. CT file of the default z-score < −2 results output from ScanFold-Fold.

10.7717/peerj.6136/supp-14 Supplemental Information 14 Table S8. RNA structure webserver scorer results of the ScanFold predicted structures compared to accepted structures. The structures and sequences were uploaded to the server as shown, and scorer was run with default settings.

10.7717/peerj.6136/supp-15 Supplemental Information 15 Raw data for the HIV-1 genome. All ScanFold analysis files needed to recapitulate results for HIV-1.

## Additional Information and Declarations

### Data Availability

The following information was supplied regarding data availability: GitHub—https://github.com/moss-lab/ScanFold RNAStructuromeDB—https://structurome.bb.iastate.edu/

## References

[ref-1] Akiyama BM, Laurence HM, Massey AR, Costantino DA, Xie X, Yang Y, Shi PY, Nix JC, Beckham JD, Kieft JS (2016). Zika virus produces noncoding RNAs using a multi-pseudoknot structure that confounds a cellular exonuclease. Science.

[ref-2] Altschul SF, Erickson BW (1985). Significance of nucleotide sequence alignments: a method for random sequence permutation that preserves dinucleotide and codon usage. Molecular Biology and Evolution.

[ref-3] Alvarez DE, Lodeiro MF, Luduena SJ, Pietrasanta LI, Gamarnik AV (2005). Long-range RNA-RNA interactions circularize the dengue virus genome. Journal of Virology.

[ref-4] Andrews RJ, Baber L, Moss WN (2017). RNAStructuromeDB: A genome-wide database for RNA structural inference. Scientific Reports.

[ref-5] Atieh T, Baronti C, De Lamballerie X, Nougairede A (2016). Simple reverse genetics systems for Asian and African Zika viruses. Scientific Reports.

[ref-6] Babak T, Blencowe BJ, Hughes TR (2007). Considerations in the identification of functional RNA structural elements in genomic alignments. BMC Bioinformatics.

[ref-7] Bellaousov S, Reuter JS, Seetin MG, Mathews DH (2013). RNAstructure: web servers for RNA secondary structure prediction and analysis. Nucleic Acids Research.

[ref-8] Bernhart SH, Hofacker IL, Stadler PF (2006). Local RNA base pairing probabilities in large sequences. Bioinformatics.

[ref-9] Busan S, Weeks KM (2017). Visualization of RNA structure models within the integrative genomics viewer. RNA.

[ref-10] Chapman EG, Moon SL, Wilusz J, Kieft JS (2014). RNA structures that resist degradation by Xrn1 produce a pathogenic Dengue virus RNA. Elife.

[ref-11] Clote P, Ferre F, Kranakis E, Krizanc D (2005). Structural RNA has lower folding energy than random RNA of the same dinucleotide frequency. RNA.

[ref-12] Coutard B, Barral K, Lichiere J, Selisko B, Martin B, Aouadi W, Lombardia MO, Debart F, Vasseur J-J, Guillemot JC, Canard B, Decroly E (2017). Zika virus methyltransferase: structure and functions for drug design perspectives. Journal of Virology.

[ref-13] Darty K, Denise A, Ponty Y (2009). VARNA: interactive drawing and editing of the RNA secondary structure. Bioinformatics.

[ref-14] Das AT, Klaver B, Berkhout B (1998). The 5′ and 3′ TAR elements of human immunodeficiency virus exert effects at several points in the virus life cycle. Journal of Virology.

[ref-15] Davis WG, Basu M, Elrod EJ, Germann MW, Brinton MA (2013). Identification of cis-acting nucleotides and a structural feature in West Nile virus 3′-terminus rna that facilitate viral minus strand RNA synthesis. Journal of Virology.

[ref-16] Deigan KE, Li TW, Mathews DH, Weeks KM (2009). Accurate SHAPE-directed RNA structure determination. Proceedings of the National Academy of Sciences of the United States of America.

[ref-17] Ding YL, Kwok CK, Tang Y, Bevilacqua PC, Assmann SM (2015). Genome-wide profiling of in vivo RNA structure at single-nucleotide resolution using structure-seq. Nature Protocols.

[ref-18] Donald CL, Brennan B, Cumberworth SL, Rezelj VV, Clark JJ, Cordeiro MT, Freitas De Oliveira Franca R, Pena LJ, Wilkie GS, Da Silva Filipe A, Davis C, Hughes J, Varjak M, Selinger M, Zuvanov L, Owsianka AM, Patel AH, McLauchlan J, Lindenbach BD, Fall G, Sall AA, Biek R, Rehwinkel J, Schnettler E, Kohl A (2016). Full genome sequence and sfRNA interferon antagonist activity of Zika virus from Recife, Brazil. PLOS Neglected Tropical Diseases.

[ref-19] Dong H, Fink K, Zust R, Lim SP, Qin C-F, Shi P-Y (2014). Flavivirus RNA methylation. Journal of General Virology.

[ref-20] Elghonemy S, Davis WG, Brinton MA (2005). The majority of the nucleotides in the top loop of the genomic 3′ terminal stem loop structure are cis-acting in a West Nile virus infectious clone. Virology.

[ref-21] Fang R, Moss WN, Rutenberg-Schoenberg M, Simon MD (2015). Probing xist RNA structure in cells using targeted structure-seq. PLOS Genetics.

[ref-22] Filomatori CV, Lodeiro MF, Alvarez DE, Samsa MM, Pietrasanta L, Gamarnik AV (2006). A 5′ RNA element promotes dengue virus RNA synthesis on a circular genome. Genes & Development.

[ref-23] Forsdyke DR (2007). Calculation of folding energies of single-stranded nucleic acid sequences: conceptual issues. Journal of Theoretical Biology.

[ref-24] Freyhult E, Gardner PP, Moulton V (2005). A comparison of RNA folding measures. BMC Bioinformatics.

[ref-25] Fu Y, Xu ZZ, Lu ZJ, Zhao S, Mathews DH (2015). Discovery of Novel ncRNA Sequences in Multiple Genome Alignments on the Basis of Conserved and Stable Secondary Structures. PLOS ONE.

[ref-26] Gesell T, Washietl S (2008). Dinucleotide controlled null models for comparative RNA gene prediction. BMC Bioinformatics.

[ref-27] Goertz GP, Abbo SR, Fros JJ, Pijlman GP (2017). Functional RNA during Zika virus infection. Virus Research.

[ref-28] Goertz GP, Fros JJ, Miesen P, Vogels CBF, Van Der Bent ML, Geertsema C, Koenraadt CJM, Van Rij RP, Van Oers MM, Pijlman GP (2016). Noncoding subgenomic flavivirus RNA is processed by the mosquito RNA interference machinery and determines West Nile virus transmission by culex pipiens mosquitoes. Journal of Virology.

[ref-29] Gokhale NS, McIntyre ABR, McFadden MJ, Roder AE, Kennedy EM, Gandara JA, Hopcraft SE, Quicke KM, Vazquez C, Willer J, Ilkayeva OR, Law BA, Holley CL, Garcia-Blanco MA, Evans MJ, Suthar MS, Bradrick SS, Mason CE, Horner SM (2016). N6-methyladenosine in Flaviviridae viral RNA genomes regulates infection. Cell Host Microbe.

[ref-30] Gruber AR, Findeiss S, Washietl S, Hofacker IL, Stadler PF (2010). RNAz 2.0: improved noncoding RNA detection. Pacific Symposium on Biocomputing.

[ref-31] Gruber AR, Neubock R, Hofacker IL, Washietl S (2007). The RNAz web server: prediction of thermodynamically stable and evolutionarily conserved RNA structures. Nucleic Acids Research.

[ref-32] Gupta AK, Kaur K, Rajput A, Dhanda SK, Sehgal M, Khan MS, Monga I, Dar SA, Singh S, Nagpal G, Usmani SS, Thakur A, Kaur G, Sharma S, Bhardwaj A, Qureshi A, Raghava GP, Kumar M (2016). ZikaVR: an integrated Zika virus resource for genomics, proteomics, phylogenetic and therapeutic analysis. Scientific Reports.

[ref-33] Huber RG, Lim XN, Ng WC, Sim A, Poh HX, Shen Y, Lim SY, Sundstrom AKB, Sun X, Aw JG, Too HK, Boey PH, Wilm A, Chawla T, Choy MJ, Jiang L, Sessions PF, Loh XJ, Alonso S, Hibberd M, Nagarajan N, Ooi EE, Bond PJ, Sessions OM, Wan Y (2018). Structure mapping of dengue and Zika viruses reveals new functional long-range interactions. bioRxiv preprint.

[ref-34] Jiang T, Nogales A, Baker SF, Martinez-Sobrido L, Turner DH (2016). Mutations designed by ensemble defect to misfold conserved RNA structures of influenza a segments 7 and 8 affect splicing and attenuate viral replication in cell culture. PLOS ONE.

[ref-35] Katoh K, Rozewicki J, Yamada KD (2017). MAFFT online service: multiple sequence alignment, interactive sequence choice and visualization. Briefings in Bioinformatics.

[ref-36] Khrustalev VV, Khrustaleva TA, Sharma N, Giri R (2017). Mutational pressure in Zika virus: local ADAR-editing areas associated with pauses in translation and replication. Frontiers in Cellular and Infection Microbiology.

[ref-37] Kieft JS, Rabe JL, Chapman EG (2015). New hypotheses derived from the structure of a flaviviral Xrn1-resistant RNA: conservation, folding, and host adaptation. RNA Biology.

[ref-38] Kuraku S, Zmasek CM, Nishimura O, Katoh K (2013). ALeaves facilitates on-demand exploration of metazoan gene family trees on MAFFT sequence alignment server with enhanced interactivity. Nucleic Acids Research.

[ref-39] Lange SJ, Maticzka D, Mohl M, Gagnon JN, Brown CM, Backofen R (2012). Global or local? Predicting secondary structure and accessibility in mRNAs. Nucleic Acids Research.

[ref-40] Lavender CA, Gorelick RJ, Weeks KM (2015). Structure-based alignment and consensus secondary structures for three HIV-related RNA genomes. PLOS Computational Biology.

[ref-41] Lichinchi G, Zhao BS, Wu Y, Lu Z, Qin Y, He C, Rana TM (2016). Dynamics of human and viral RNA methylation during Zika virus infection. Cell Host & Microbe.

[ref-42] Lim CS, Brown CM (2017). Know your enemy: successful bioinformatic approaches to predict functional RNA structures in viral RNAs. Frontiers in Microbiology.

[ref-43] Liu ZY, Li XF, Jiang T, Deng YQ, Zhao H, Wang HJ, Ye Q, Zhu SY, Qiu Y, Zhou X, Qin ED, Qin CF (2013). Novel cis-acting element within the capsid-coding region enhances flavivirus viral-RNA replication by regulating genome cyclization. Journal of Virology.

[ref-44] Lodeiro MF, Filomatori CV, Gamarnik AV (2009). Structural and functional studies of the promoter element for dengue virus RNA replication. Journal of Virology.

[ref-45] Lorenz R, Bernhart SH, Honer Zu Siederdissen C, Tafer H, Flamm C, Stadler PF, Hofacker IL (2011). ViennaRNA Package 2.0. Algorithms for Molecular Biology.

[ref-46] Markham NR, Zuker M (2008). UNAFold: software for nucleic acid folding and hybridization. Methods in Molecular Biology.

[ref-47] Mathews DH (2004). Using an RNA secondary structure partition function to determine confidence in base pairs predicted by free energy minimization. RNA.

[ref-48] Mathews DH, Disney MD, Childs JL, Schroeder SJ, Zuker M, Turner DH (2004). Incorporating chemical modification constraints into a dynamic programming algorithm for prediction of RNA secondary structure. Proceedings of the National Academy of Sciences of the United States of America.

[ref-49] Mathews DH, Moss WN, Turner DH (2010). Folding and finding RNA secondary structure. Cold Spring Harbor Perspectives in Biology.

[ref-50] Mathews DH, Sabina J, Zuker M, Turner DH (1999). Expanded sequence dependence of thermodynamic parameters improves prediction of RNA secondary structure. Journal of Molecular Biology.

[ref-51] McCaskill JS (1990). The equilibrium partition function and base pair binding probabilities for RNA secondary structure. Biopolymers.

[ref-52] Mortimer SA, Trapnell C, Aviran S, Pachter L, Lucks JB (2012). SHAPE-seq: high-throughput RNA structure analysis. Current Protocols in Chemical Biology.

[ref-53] Moss WN (2018). RNA2DMut: a web tool for the design and analysis of RNA structure mutations. RNA.

[ref-54] Moss WN, Priore SF, Turner DH (2011). Identification of potential conserved RNA secondary structure throughout influenza a coding regions. RNA.

[ref-55] Moss WN, Steitz JA (2013). Genome-wide analyses of Epstein-Barr virus reveal conserved RNA structures and a novel stable intronic sequence RNA. BMC Genomics.

[ref-56] Nicholson BL, White KA (2014). Functional long-range RNA–RNA interactions in positive-strand RNA viruses. Nature Reviews Microbiology.

[ref-57] Ouellet DL, Plante I, Landry P, Barat C, Janelle ME, Flamand L, Tremblay MJ, Provost P (2008). Identification of functional microRNAs released through asymmetrical processing of HIV-1 TAR element. Nucleic Acids Research.

[ref-58] Pijlman GP, Funk A, Kondratieva N, Leung J, Torres S, Van Der Aa L, Liu WJ, Palmenberg AC, Shi PY, Hall RA, Khromykh AA (2008). A highly structured, nuclease-resistant, noncoding RNA produced by flaviviruses is required for pathogenicity. Cell Host & Microbe.

[ref-59] Pratt CA, Mowry KL (2013). Taking a cellular road-trip: mRNA transport and anchoring. Current Opinion in Cell Biology.

[ref-60] Puton T, Kozlowski LP, Rother KM, Bujnicki JM (2014). CompaRNA: a server for continuous benchmarking of automated methods for RNA secondary structure prediction. Nucleic Acids Research.

[ref-61] Reuter JS, Mathews DH (2010). RNAstructure: software for RNA secondary structure prediction and analysis. BMC Bioinformatics.

[ref-62] Ritchey LE, Su Z, Tang Y, Tack DC, Assmann SM, Bevilacqua PC (2017). Structure-seq2: sensitive and accurate genome-wide profiling of RNA structure in vivo. Nucleic Acids Research.

[ref-63] Schlick T, Pyle AM (2017). Opportunities and challenges in RNA structural modeling and design. Biophysical Journal.

[ref-64] Smyth RP, Schlub TE, Grimm AJ, Waugh C, Ellenberg P, Chopra A, Mallal S, Cromer D, Mak J, Davenport MP, Hahn BH (2014). Identifying recombination hot spots in the HIV-1 genome. Journal of Virology.

[ref-65] Stockley PG, Twarock R, Bakker SE, Barker AM, Borodavka A, Dykeman E, Ford RJ, Pearson AR, Phillips SE, Ranson NA, Tuma R (2013). Packaging signals in single-stranded RNA viruses: nature’s alternative to a purely electrostatic assembly mechanism. Journal of Biological Physics.

[ref-66] Thiel BC, Ochsenreiter R, Gadekar VP, Tanzer A, Hofacker IL (2018). RNA structure elements conserved between mouse and 59 other vertebrates. Genes.

[ref-67] Thorvaldsdottir H, Robinson JT, Mesirov JP (2013). Integrative genomics viewer (IGV): high-performance genomics data visualization and exploration. Briefings in Bioinformatics.

[ref-68] Thurner C, Witwer C, Hofacker IL, Stadler PF (2004). Conserved RNA secondary structures in Flaviviridae genomes. Journal of General Virology.

[ref-69] Villordo SM, Alvarez DE, Gamarnik AV (2010). A balance between circular and linear forms of the dengue virus genome is crucial for viral replication. RNA.

[ref-70] Villordo SM, Carballeda JM, Filomatori CV, Gamarnik AV (2016). RNA structure duplications and flavivirus host adaptation. Trends in Microbiology.

[ref-71] Washietl S (2007). Prediction of structural noncoding RNAs with RNAz. Methods in Molecular Biology.

[ref-72] Washietl S, Hofacker IL, Lukasser M, Huttenhofer A, Stadler PF (2005a). Mapping of conserved RNA secondary structures predicts thousands of functional noncoding RNAs in the human genome. Nature Biotechnology.

[ref-73] Washietl S, Hofacker IL, Stadler PF (2005b). Fast and reliable prediction of noncoding RNAs. Proceedings of the National Academy of Sciences of the United States of America.

[ref-74] Washietl S, Hofacker IL, Stadler PF, Kellis M (2012). RNA folding with soft constraints: reconciliation of probing data and thermodynamic secondary structure prediction. Nucleic Acids Research.

[ref-75] Watts JM, Dang KK, Gorelick RJ, Leonard CW, Bess JW, Swanstrom R, Burch CL, Weeks KM (2009). Architecture and secondary structure of an entire HIV-1 RNA genome. Nature.

[ref-76] Wilkinson KA, Gorelick RJ, Vasa SM, Guex N, Rein A, Mathews DH, Giddings MC, Weeks KM (2008). High-throughput SHAPE analysis reveals structures in HIV-1 genomic RNA strongly conserved across distinct biological states. PLOS Biology.

[ref-77] Wilkinson KA, Merino EJ, Weeks KM (2006). Selective 2′-hydroxyl acylation analyzed by primer extension (SHAPE): quantitative RNA structure analysis at single nucleotide resolution. Nature Protocols.

[ref-78] Will S, Joshi T, Hofacker IL, Stadler PF, Backofen R (2012). LocARNA-P: accurate boundary prediction and improved detection of structural RNAs. RNA.

[ref-79] Wimmer J, Fujinaga K, Taube R, Cujec TP, Zhu YR, Peng JM, Price DH, Peterlin BM (1999). Interactions between Tat and TAR and human immunodeficiency virus replication are facilitated by human cyclin T1 but not cyclins T2a or T2b. Virology.

[ref-80] Wu X, Brewer G (2012). The regulation of mRNA stability in mammalian cells: 2.0. Gene.

[ref-81] Ye Q, Liu Z-Y, Han J-F, Jiang T, Li X-F, Qin C-F (2016). Genomic characterization and phylogenetic analysis of Zika virus circulating in the Americas. Infection, Genetics and Evolution.

[ref-82] Yu L, Markoff L (2005). The topology of bulges in the long stem of the flavivirus 3′ stem-loop is a major determinant of RNA replication competence. Journal of Virology.

[ref-83] Zarringhalam K, Meyer MM, Dotu I, Chuang JH, Clote P (2012). Integrating chemical footprinting data into RNA secondary structure prediction. PLOS ONE.

[ref-84] Zeng LL, Falgout B, Markoff L (1998). Identification of specific nucleotide sequences within the conserved 3′-SL in the dengue type 2 virus genome required for replication. Journal of Virology.

