# Supplementary figures and images for: ScanFold: an approach for genome-wide discovery of local RNA structural elements—applications to Zika virus and HIV
Source: PeerJ. 2018 Dec 18;6:e6136. doi: 10.7717/peerj.6136 (PMC6317755; doi:10.7717/peerj.6136)

**a**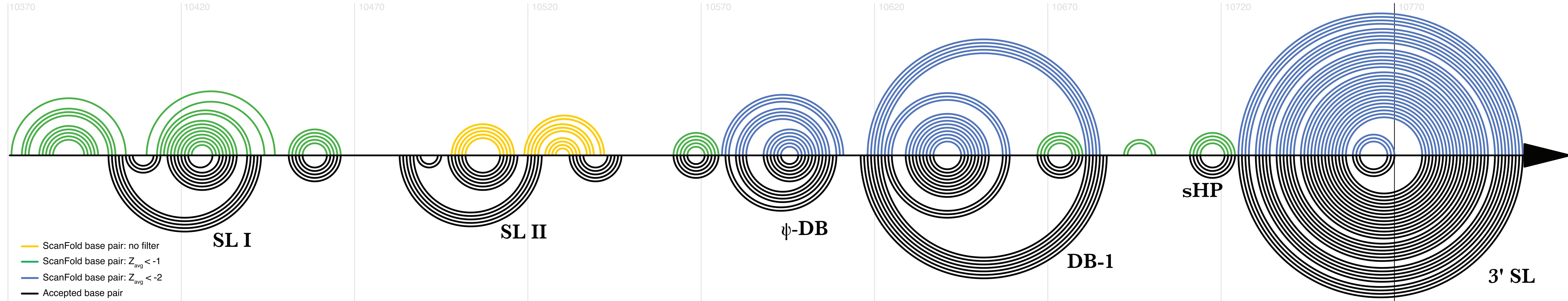

Supplement: Supplemental Information 2 — (a) Arc diagram of the 5′ end region as predicted via ScanFold-Fold; base pairs are colored by their z-score cutoff where blue lines depict base pairs which were predicted in the z-score < −2 results (Table S7) and green lines refer to base pairs which were predicted in the z-score < −1 results (Table S6). (b) Arc diagram of the accepted secondary structure model for the 5′ end of ZIKV as shown in (Ye et al., 2016) and mapped to the KJ776791.2 sequence. [file peerj-06-6136-s002.pdf]

**a**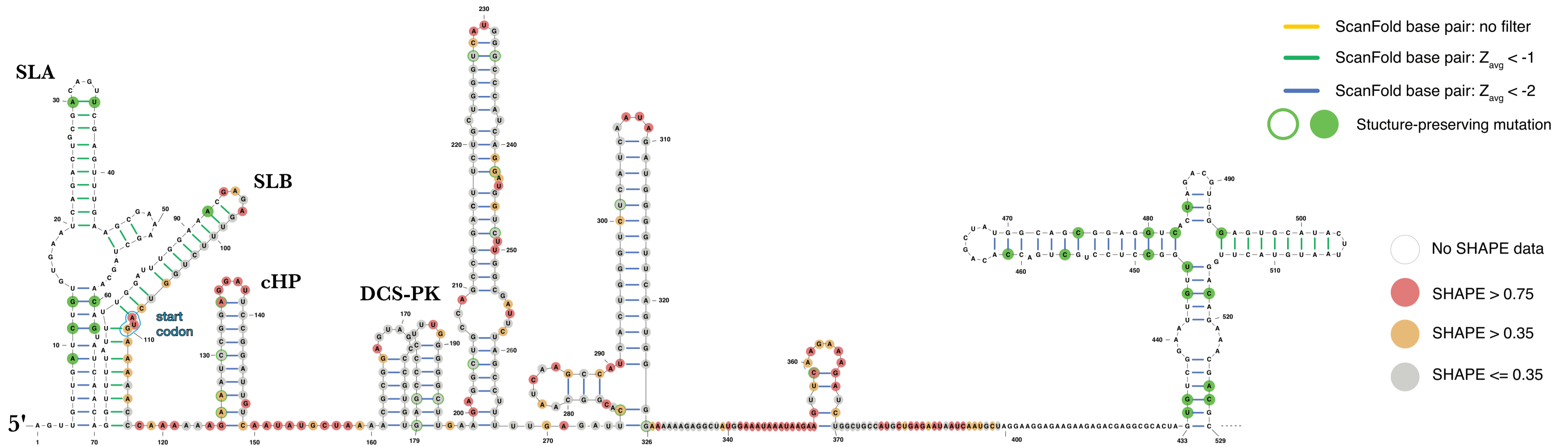**b**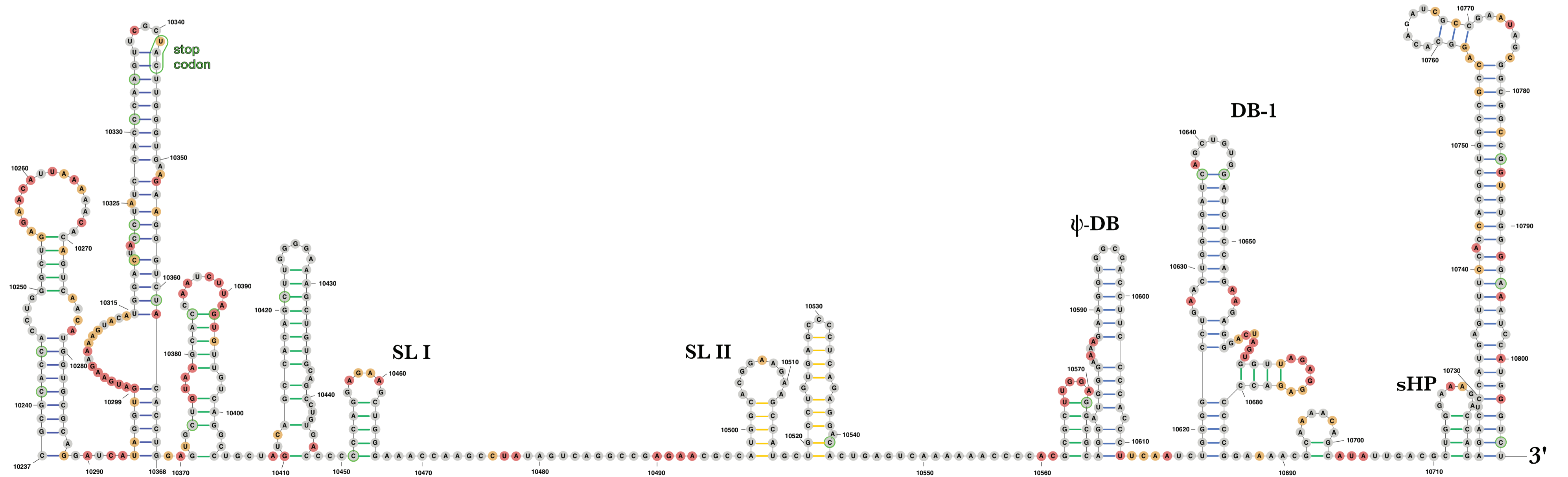

Supplement: Supplemental Information 4 — Base pairs are colored by their z-score cutoff: blue lines depict base pairs which were predicted in the z-score < −2 results (Table S7), green lines refer to base pairs which were predicted in the z-score < −1 results (Table S6), and yellow lines were predicted in the no filter results (Table S5). The start and stop codon nucleotides have been circled and labeled in blue and green respectively. Nucleotides which established ScanFold base pair preserving mutations within the alignment are highlighted with filled green circles. The SHAPE reactivity scores available from dataset 6 of (Huber et al., 2018) have been mapped onto nucleotides (where data is available). [file peerj-06-6136-s004.pdf]

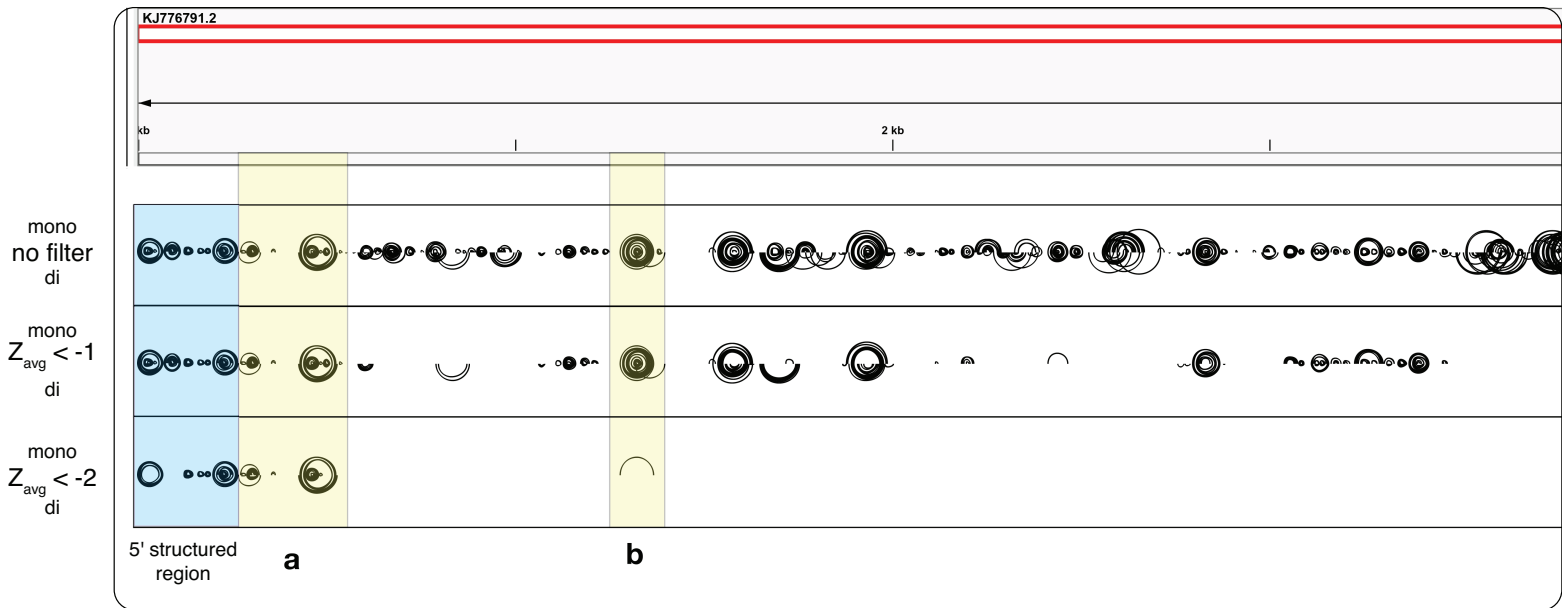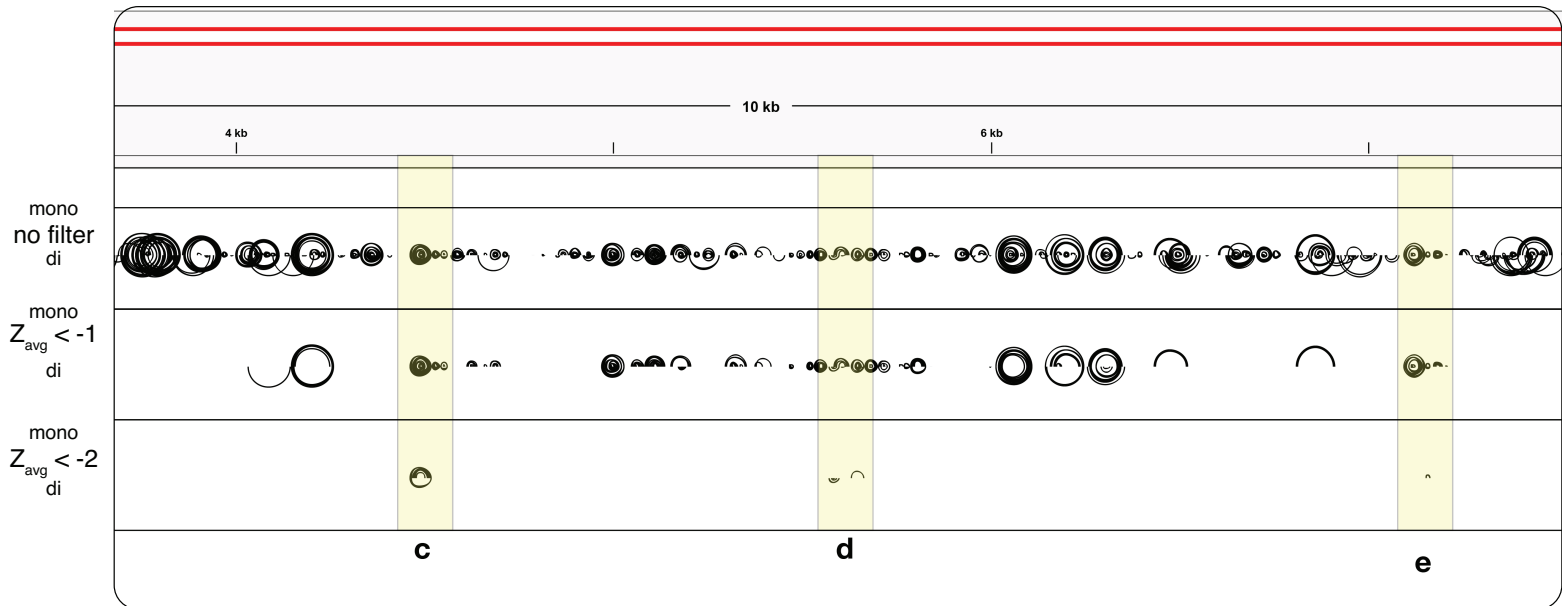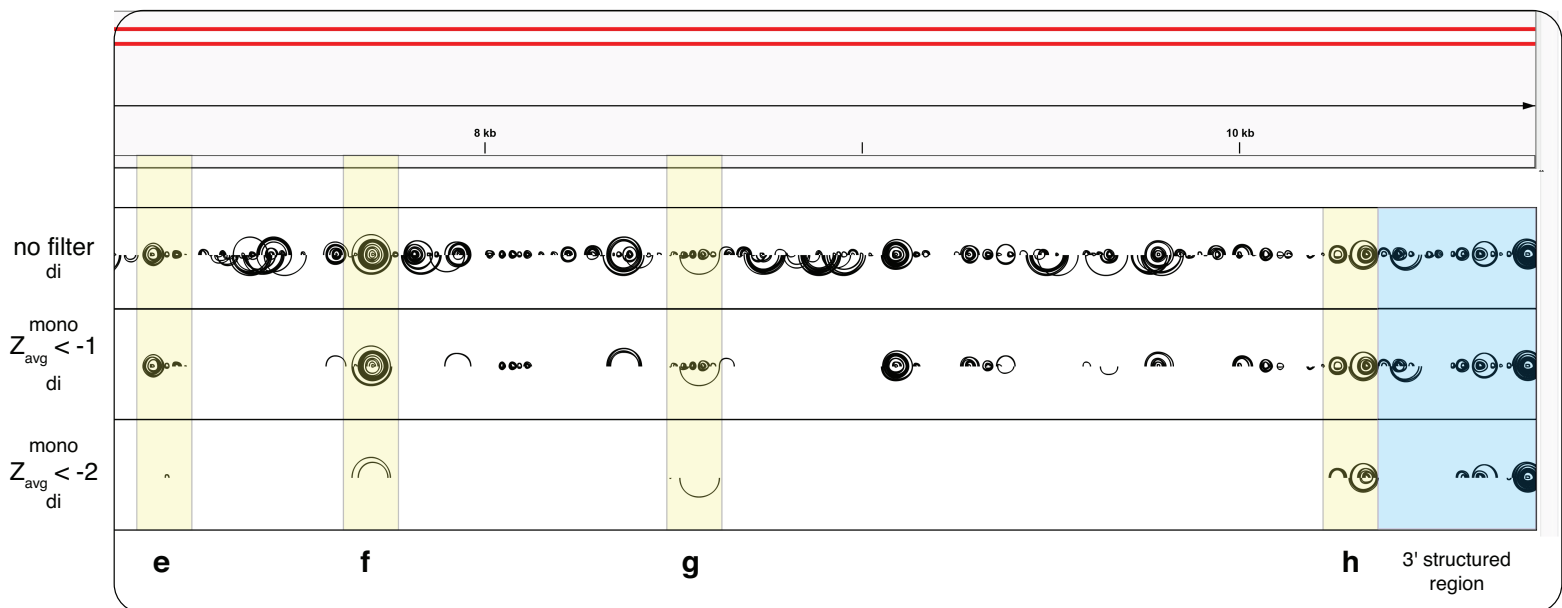

Supplement: Supplemental Information 5 — The entirety of base pairs predicted throughout the genome of ZIKV have been plotted as RNA base pairing tracks using IGV. Three Zavg filter values were used and plotted as separate tracks (labeled as such). Each of these tracks depicts results when using a mononucleotide (top) or dinucleotide (bottom) shuffling technique. The known structured regions on the 5′ and 3′ end have been highlighted in blue. The novel structures predicted (with Zavg scores < −2) in the core coding region have been highlighted in yellow and labeled (a) to (h) based on their genomic location. [file peerj-06-6136-s005.pdf]

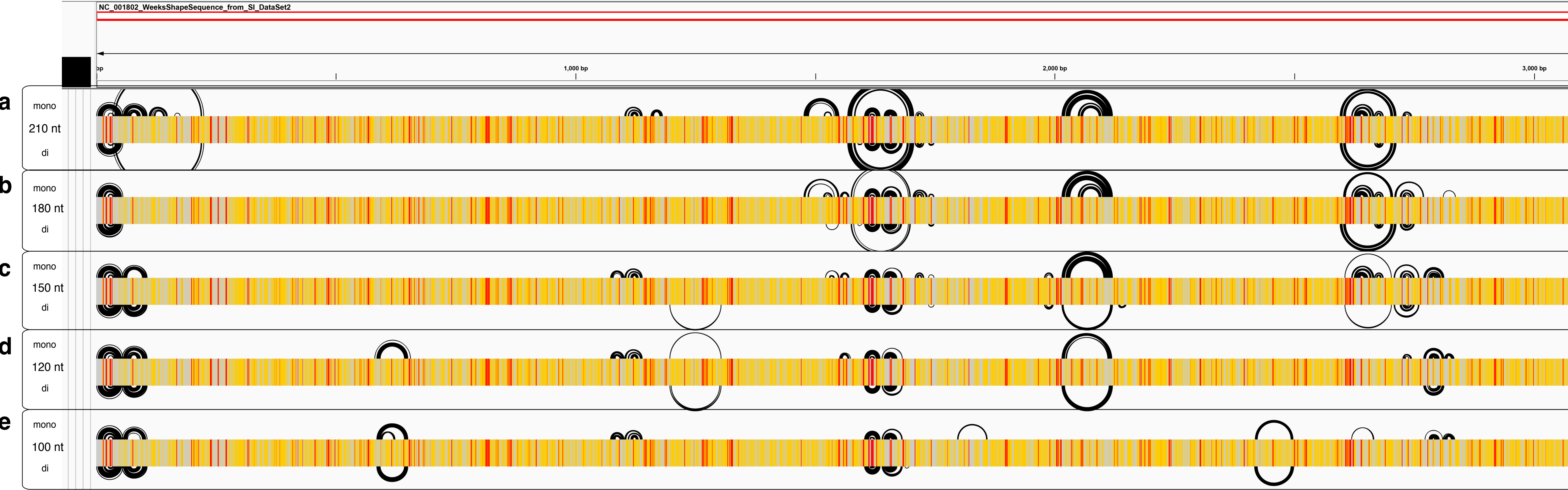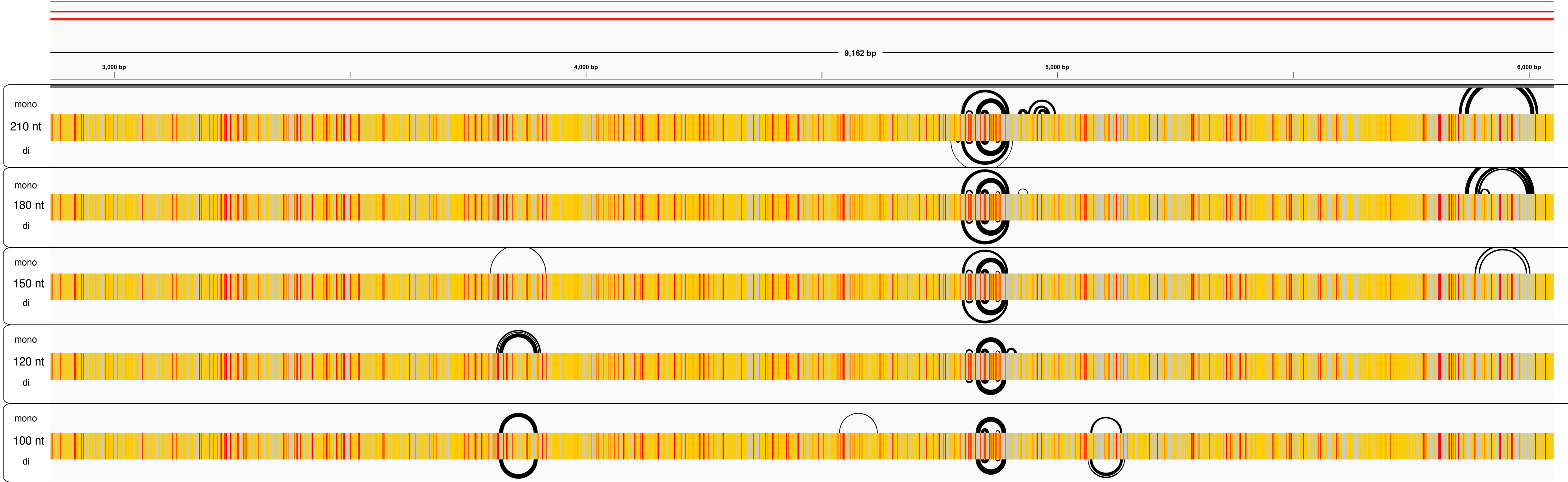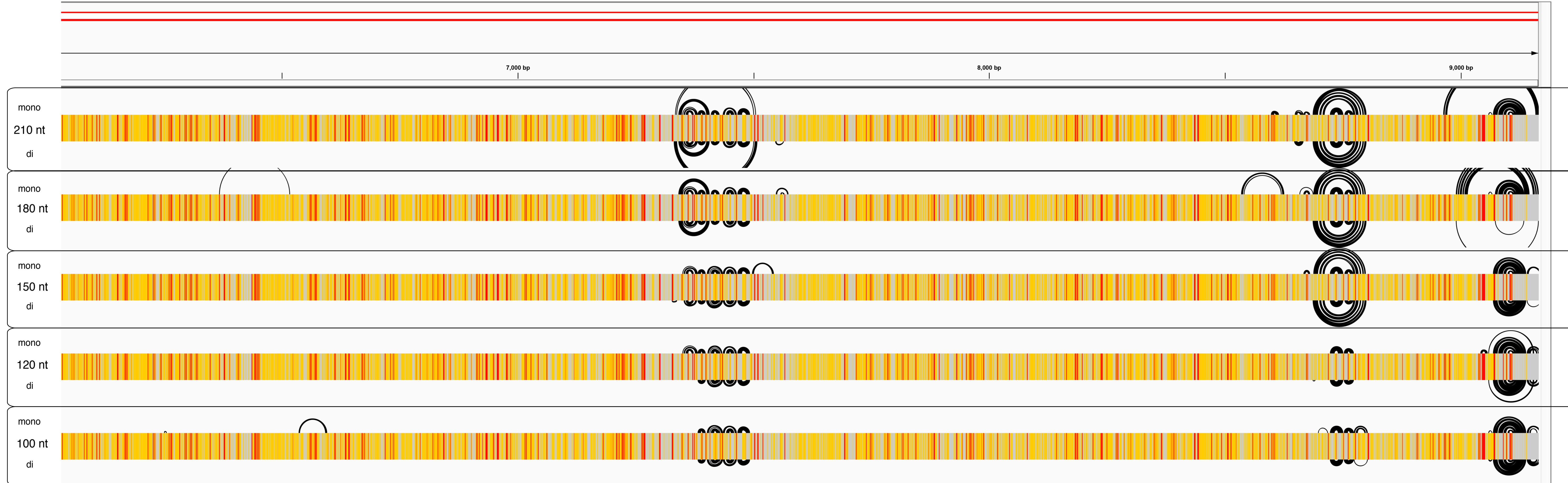

Supplement: Supplemental Information 6 — Here, the complete set of ScanFold-Fold Zavg < −2 base pairs generated across the HIV-1 genome are depicted as RNA base pairing tracks (Busan & Weeks, 2017) on IGV (Thorvaldsdottir, Robinson & Mesirov, 2013). Each of these tracks depicts results when using either a mononucleotide (top) or dinucleotide (bottom) shuffling technique to calculate the z-score. Each of the five tracks shows results when using a different window size: (a) 210 nt (b) 180 nt (c) 150 nt (d) 120 nt (the default window size) and (e) 100 nt. SHAPE reactivity data from (Watts et al., 2009) is shown as a heat map for each track where a reactivity < = 0.35 is colored grey, a reactivity > 0.75 is red and a reactivity between 0.35 and 0.75 is yellow. [file peerj-06-6136-s006.pdf]
