# Supplementary material for: ScanFold: an approach for genome-wide discovery of local RNA structural elements—applications to Zika virus and HIV
Source: PeerJ. 2018 Dec 18;6:e6136. doi: 10.7717/peerj.6136 (PMC6317755; doi:10.7717/peerj.6136)
Supplement: Supplemental Information 3 — (a) Arc diagram of the 3′ end region as predicted via ScanFold; base pairs are colored by their z-score cutoff where blue lines depict base pairs which were predicted in the z-score < −2 results (Table S7), green lines refer to base pairs which were predicted in the z-score < −1 results (Table S6), and yellow lines were predicted in the no filter results (Table S5). (b) Arc diagram of the accepted secondary structure model for the 3′ end of ZIKV as shown in (Goertz et al., 2017) mapped to the KJ776791.2 sequence. The start codon nucleotide locations have been highlighted with a light blue bar. [file peerj-06-6136-s003.pdf]

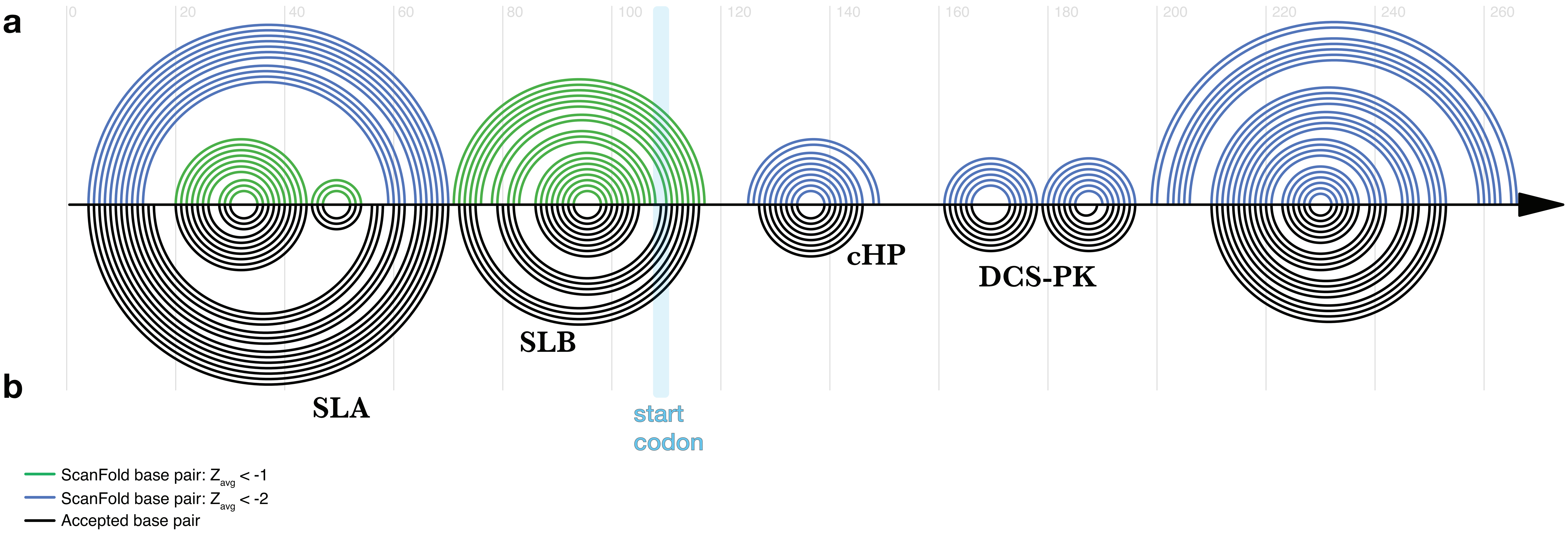

a
b
0
20
40
60
80
100
120
140
160
180
200
220
240
260
SLA
SLB
cHP
DCS-PK
start
codon
ScanFold base pair: Z avg < -1
ScanFold base pair: Z avg < -2
Accepted base pair
