# Supplementary material for: ScanFold: an approach for genome-wide discovery of local RNA structural elements—applications to Zika virus and HIV
Source: PeerJ. 2018 Dec 18;6:e6136. doi: 10.7717/peerj.6136 (PMC6317755; doi:10.7717/peerj.6136)
Supplement: Supplemental Information 1 — This image depicts the base pairs identified on the full length of the ZIKV genome (accession KJ776791.2) through each step of ScanFold-Fold processing, as base pairing tracks (Busan & Weeks, 2017) on IGV (Thorvaldsdottir, Robinson & Mesirov, 2013). (a) The first track shows the totality of base pairs predicted throughout the ScanFold-Scan process. (b) The second track depicts the base pairs which remain after ScanFold-Fold selects the most favorable base pair (according to the lowest Znorm; see Methods Eq. 3) per i nucleotide of the sequence; competition is allowed, whereby multiple partners are permitted to pair with the same nucleotides. (c) The third track shows the base pairs which remain after prohibiting multiple pairing partners per nucleotide; competition is disallowed, i.e. for nucleotides predicted to have more than one most favorable partner, only a single pairing partner is allowed, selected as the one with the lowest Znorm. This track is equivalent to the “no filter” results from ScanFold-Fold. The base pairs from this track are then subjected to filtering based on their Zavg (see Methods Eq. 2). The final tracks depict which base pairs from the results above, generated Zavg scores less than (d) −1 (e) −1.6 (one standard deviation below the mean z-score) and (f) −2. [file peerj-06-6136-s001.pdf]

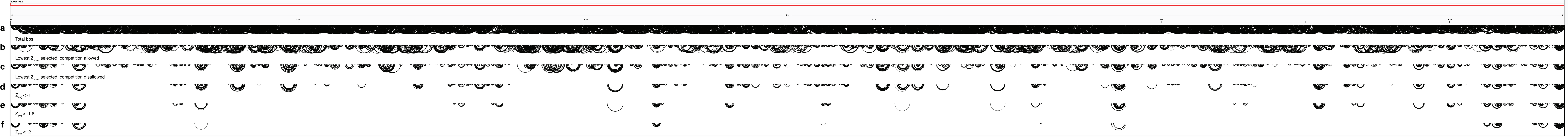
KJ776791.2
10 kb
kb
6 kb
a
Total bps
b
Lowest Z_norm selected; competition allowed
c
Lowest Z_norm selected; competition disallowed
d
Z_avg < -1
e
Z_avg < -1.6
f
Z_avg < -2
